# Hypermethylation of the *Bmp4* promoter dampens binding of HIF-1α and impairs its cardiac protective effects from oxidative stress in prenatally GC-exposed offspring

**DOI:** 10.1007/s00018-023-04703-0

**Published:** 2023-02-06

**Authors:** Ling-Tong Gao, Jian-Qiang Yuan, Zhi-Yu Zhang, Hou-Ming Zhao, Lu Gao

**Affiliations:** 1 Department of Physiology, Naval Medical University, 800 Xiangyin Rd., Shanghai, 200433 People’s Republic of China; 2 Department of Health Management, Changzheng Hospital, Naval Medical University, Shanghai, 200003 People’s Republic of China; 3 Shanghai Key Laboratory for Assisted Reproduction and Reproductive Genetics, Shanghai, People’s Republic of China; 4 Shanghai Key Laboratory of Embryo Original Diseases, Shanghai, People’s Republic of China

**Keywords:** BMP4, HIF-1α, DNA methylation, Cardiac function, Mitophagy, Glucocorticoid, Programming effect

## Abstract

The exposure to an unhealthy environment in utero can lead to the occurrence of cardiovascular diseases in the offspring. Glucocorticoids (GC) are essential for normal development and maturation of fetal organs and is a first-line treatment for pregnant women affected by autoimmune diseases. However, excess prenatal GC exposure might program the development of fetal organs and cause a number of chronic diseases in later life. Our previous studies indicated that cardiac functions were significantly compromised in rat offspring prenatally exposed to the synthetic glucocorticoid dexamethasone (DEX), only after ischemia–reperfusion. In the present study, we further observed that DNA hypermethylation of bone morphogenetic protein 4 (*Bmp4*) promoter in cardiomyocytes caused by prenatal DEX exposure substantially dampened the binding activity of transcription factor HIF-1α induced by cardiac ischemia. Therefore, prenatal DEX exposure inhibits the induction of BMP4 upon *I*/*R* and attenuates the protective effects of BMP4 in cardiomyocytes, which eventually manifests as malfunction of the adult heart. Moreover, we employed two cardiac-specific *Bmp4* knock-in mouse models and found that in vivo BMP4 overexpression could rescue the cardiac dysfunction caused by prenatal GC exposure. In depth mechanistic research revealed that BMP4 protects the cardiomyocytes from mitophagy and apoptosis by attenuating mitochondrial PGC-1α expression in a p-Smad and Parkin-dependent manner. These findings suggest that prenatal GC exposure increases the susceptibility of the offspring’s heart to a “second strike” after birth, due to the failure of hypoxia-induced HIF-1α transactivation of the hypermethylated *Bmp4* promoter in cardiomyocytes. Pretreatment with the DNA methylation inhibitor, 5-Aza-2′-deoxycytidine, could be a potential therapeutic method for this programming effect of GC exposure during pregnancy on neonatal cardiac dysfunction.

**Supplementary Information:**

The online version contains supplementary material available at 10.1007/s00018-023-04703-0.

## Introduction

Among non-communicable diseases, cardiovascular diseases (CVDs) are the major cause of morbidity and mortality worldwide [1]. In recent years, accumulating evidence indicates that exposure to an unhealthy environment in utero can lead to the occurrence of CVDs in the offspring, even into adulthood. Previously, Barker et al. discovered the association of an adverse intrauterine environment with an increased risk of hypertension and ischemic heart disease in adulthood [2]. This is now referred to as the Developmental Origins of Health and Disease (DOHAD). Thereafter, more and more influences of the intrauterine environment on CVDs have been discovered. Maternal glucocorticoid (GC) overexposure [3, 4], preeclampsia, diabetes [5–7], maternal obesity or excessive weight gain during pregnancy [8, 9], as well as maternal undernutrition [10], tobacco smoking [11, 12], maternal stress or alcohol consumption [13] during pregnancy, all greatly enhance risks of CVDs for offspring in their later life. Therefore, scientists worldwide have tried to answer the question of how to mitigate the cardiac damages caused by risk factors within the intrauterine environment.

Endogenous GC provides a critical developmental trigger, which is essential for normal development and maturation of many organs, including thyroid, kidney, brain, pituitary, and the fetal lung [14–16]. This makes GC a widely used therapy for women at risk of preterm birth to accelerate the maturation of fetal organs. Meanwhile, GC is also a first-line and compulsory treatment for pregnant women with asthma [17] or an autoimmune disease [18, 19], throughout the entire pregnancy until labor.

However, prenatal exposure to excess GC might cause a number of chronic diseases in later life, including diabetes mellitus, obesity, psychiatric diseases and cardiovascular diseases [15, 20]. In our recent study, we found that the cardiac functions were significantly compromised in male rat offspring prenatally exposed to the synthetic glucocorticoid dexamethasone (DEX) compared to those exposed to normal saline (NS), due to an increased infarction and apoptosis of myocardium, especially after myocardial ischemia and reperfusion injury (IRI) [21]. MeDIP-sequencing analysis revealed that the regions of bone morphogenetic protein 4 (*Bmp4*) promoter were hypermethylated due to prenatal exposure to DEX, which subsequently lead to the decreased Bmp4 expression [21]. The cardiac dysfunction was associated with a breakdown of mitochondrial membrane potential, a decrease in ATP production and the dramatically elevated release of reactive oxygen species (ROS), which could be rescued by the addition of exogenous Bmp4 [21]. However, why the cardiac functions were abnormal in the DEX-exposed offspring only after IRI has not been elucidated.

BMP4 is a secretory protein which belongs to transforming growth factor-beta (TGF-β) superfamily [22]. BMP4 is required for the development of the atrioventricular canal [23], branchial-arch artery remodeling [24], endocardial cushion development and for normal separation of the outflow tract [25]. Although BMP4 is very important for heart development during embryogenesis, it is almost undetectable in the adult heart [26]. However, BMP4 levels are elevated with myocardial injury in adult humans or mice. Plasma BMP4 levels in heart failure patients are found to be significantly higher in heart failure patients than that of subjects without heart failure [26]. *Bmp4* mRNA was increased in left ventricular biopsies of patients with coronary artery disease with good cardiac function [27]. BMP4 expression was markedly increased in mouse hearts after 2- and 4-week transverse aortic constriction (TAC) [26] or 24 h post-infarction by permanent coronary artery ligation [27]. All of these findings suggest that BMP4 may exert protective effects against myocardial ischemic injury. Signaling of BMP4 through BMP receptors (BmpRI and BmpRII) is involved in two pathways: Smad and non-Smad pathways [28, 29], whereas the signaling pathway and molecular mechanisms underlying the protective effect of BMP4 in IRI have yet been fully illustrated.

In the present study, we aimed to investigate the protective effects and underlying signaling pathways of BMP4 on myocardium in vivo, and to dissect the molecular mechanisms mediating the upregulation of BMP4 under hypoxia which is attenuated by the prenatal GC exposure. We employed a cardiac specific *Bmp4* knock-in mouse model in which BMP4 expression was specifically induced in the myocardium and found that BMP4 could protect the cardiomyocytes from apoptosis by attenuating mitochondrial PGC-1α expression in a p-Smad and Parkin-dependent manner. The ischemia-induced transcription factor HIF-1α bound to the *Bmp4* promoter and activated *Bmp4* transcription. DEX treatment caused the hypermethylation of HIF-1α binding sites on the *Bmp4* promoter and impaired the transactivation of *Bmp4*, which led to the failure of increase in BMP4 upon IRI and attenuates the protective effects of BMP4 in the cardiomyocytes, and eventually manifests the malfunction of adult heart.

## Results

### GC exposure during pregnancy causes hypermethylation of the *Bmp4* promoter which inhibits transactivation by HIF-1α in offspring’s cardiomyocytes

**Fig. 1:**
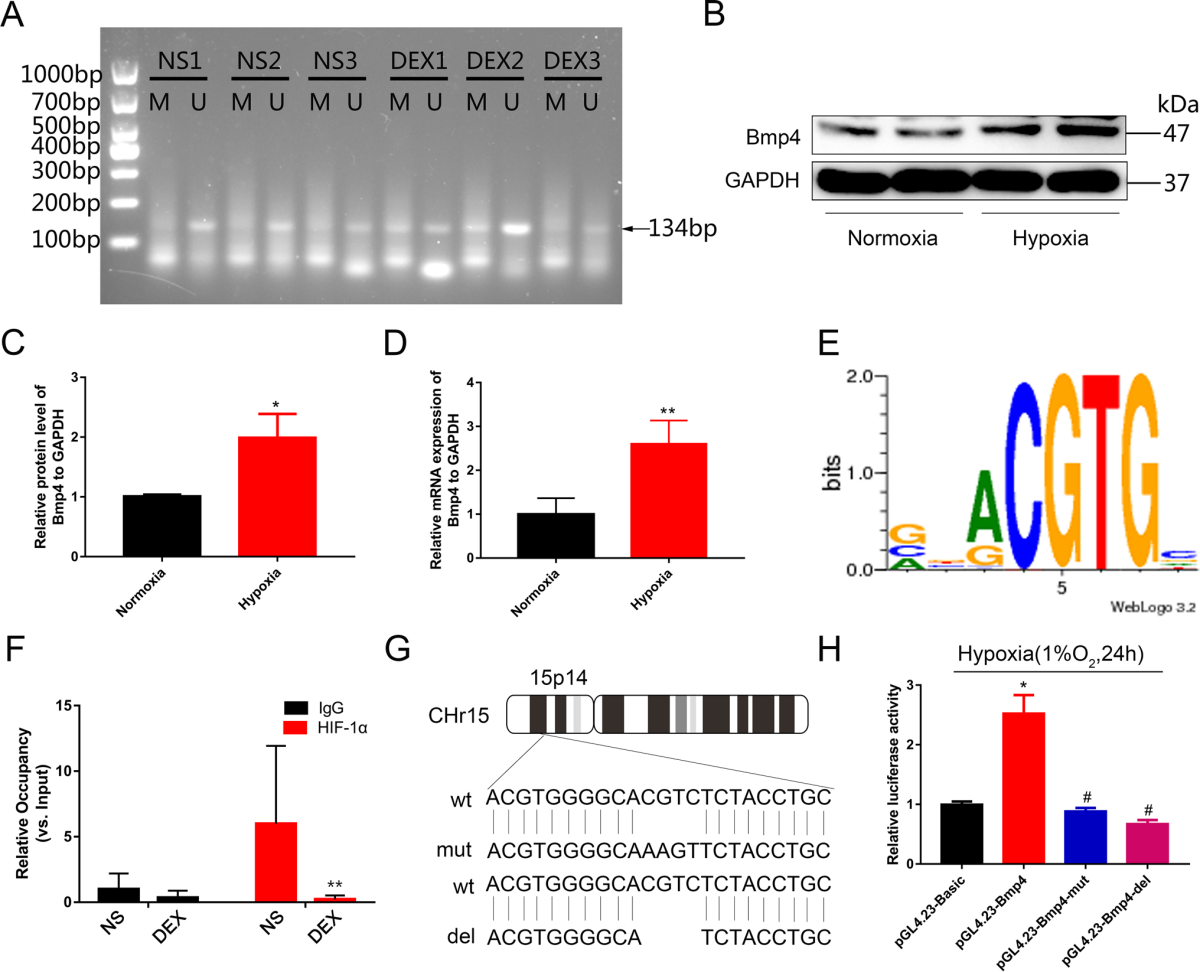
Excess GC exposure during pregnancy causes hypermethylation of the *Bmp4* promoter; this inhibits transactivation by HIF-1α in cardiomyocytes of male rat offspring. The DNA methylation level of the *Bmp4* promoter in cardiomyocytes of male rat offspring was analyzed by methylation-specific PCR, and the methylated PCR product and unmethylated PCR product was labeled as M and U, respectively (**A**, *n* = 3 per treatment group). Protein levels of BMP4 in NRCMs were significantly increased upon *H*/*R* treatment compared with normoxia treatment. Representative Western blot (**B**) and the corresponding histogram (**C**) are shown separately. *n* = 5 per treatment group, *p < 0.05, compared with normoxia treatment. mRNA levels of *Bmp4* in NRCMs were also significantly increased upon *H*/*R* treatment (**D**). *n* = 5, **p < 0.01, compared with normoxia treatment. Bioinformatics prediction of the HIF-1α binding site on *Bmp4* promoter was presented in panel E. The putative binding sequence was 5′-GCACGTCT-3′ (**E**). The binding ability of HIF-1α to the *Bmp4* promoter upon *I*/*R* treatment was significantly decreased in the myocardium of rat offspring prenatally exposed to dexamethasone (DEX, **F**). *n* = 4, **p < 0.01, compared with rat offspring prenatally exposed to normal saline (NS). The putative binding site as well as the core sequences for DNA methylation on *Bmp4* promoter were mutated or deleted (**G**). The transcriptional activity of luciferase was significantly increased after pGL4.23-*Bmp4* transfection into H9C2 cells upon hypoxia treatment, which was diminished by the transfection of pGL4.23-*Bmp4*-mut or pGL4.23-*Bmp4*-del plasmids (H). *n* = 3, *p < 0.05, compared with pGL4.23-basic plasmid transfection; #*p* < 0.05, compared with pGL4.23-*Bmp4* plasmid transfection

In a previous MeDIP-sequencing analysis, it was found that the methylation level of the *Bmp4* promoter in cardiomyocytes of prenatally GC-exposed male offspring was significantly increased, along with the decrease in mRNA and protein expression of Bmp4 [21]. To verify effects of exposure of male rat offspring to DEX *vs.* NS during pregnancy on DNA methylation of the *Bmp4* promoter, genomic DNA was extracted from myocardium and analyzed using MSP. The results showed that the DNA methylation level of *Bmp4* promoter was significantly higher in the DEX-treated group compared to that treated with NS, indicated by the increased ratio of methylated PCR product (M) vs. unmethylated PCR product (U) between DEX-treated group and NS-treated group (Fig. 1A). In accord with our previous findings, cardiac function (indicated by ± d*p*/d*t* max) of adult offspring manifested no significant differences between DEX and NS group, but was significantly decreased in adult male rats after ischemia–reperfusion (*I*/*R*) of myocardium of the DEX-treated, compared with the NS group [21]. Therefore, we speculated that hypoxia might cause an increase in Bmp4 expression, which was attenuated by the promoter hypermethylation due to prenatal DEX exposure.

To verify this hypothesis, we analyzed the mRNA and protein expression of Bmp4 in neonatal rat cardiomyocytes (NRCMs) treated with hypoxia/reoxygenation (*H*/*R*) or under normoxic conditions. It was shown that both mRNA and protein levels of Bmp4 were significantly increased upon *H*/*R* exposure (Fig. 1B–D). Hypoxia can significantly increase expression of transcription factor HIF-1α, which binds the promoter regions and activate transcription of target genes. We employed the LASAGNA online tool to analyze the promoter region of *Bmp4* and found a putative binding site for HIF-1α within the methylation area of *Bmp4* promoter (Supplemental Table 1, Fig. 1E). ChIP-qPCR analysis showed that HIF-1α was significantly enriched in *Bmp4* promoter region in the myocardium upon *I*/*R* from prenatally NS-exposed rat offspring, while the binding ability of HIF-1α to the *Bmp4* promoter region upon *I*/*R* was significantly decreased in the myocardium of rat offspring prenatally exposed to DEX (Fig. 1F).

We next asked whether DNA hypermethylation of the *Bmp4* promoter affects the binding of HIF-1α and subsequently attenuates *Bmp4* transcriptional activity. To assess this, a dual luciferase reporter activity experiment was conducted. The promoter region of *Bmp4* containing binding sites for HIF-1α was amplified from genomic DNA and subcloned into pGL4.23 plasmid to construct pGL4.23-Bmp4 vector. Meanwhile, pGL4.23-Bmp4-mut and pGL4.23-Bmp4-del vectors were also constructed by mutating and deleting the hypermethylation core sequences of *Bmp4* promoter region, respectively (Fig. 1G). We observed that activation of HIF-1α by exposure to 1% O_2_ for 24 h significantly increased the transcriptional activity of luciferase in H9C2 cells transfected with pGL4.23-Bmp4 plasmid, compared with those transfected with a control vector lacking the *Bmp4* promoter (Fig. 1H). However, the increased transcriptional activity was blunted by transfection of pGL4.23-Bmp4-mut or pGL4.23-Bmp4-del vectors (Fig. 1H).

### Myocardial-specific BMP4 overexpression protects cardiac functions in male offspring of mice exposed to DEX during pregnancy

**Fig. 2:**
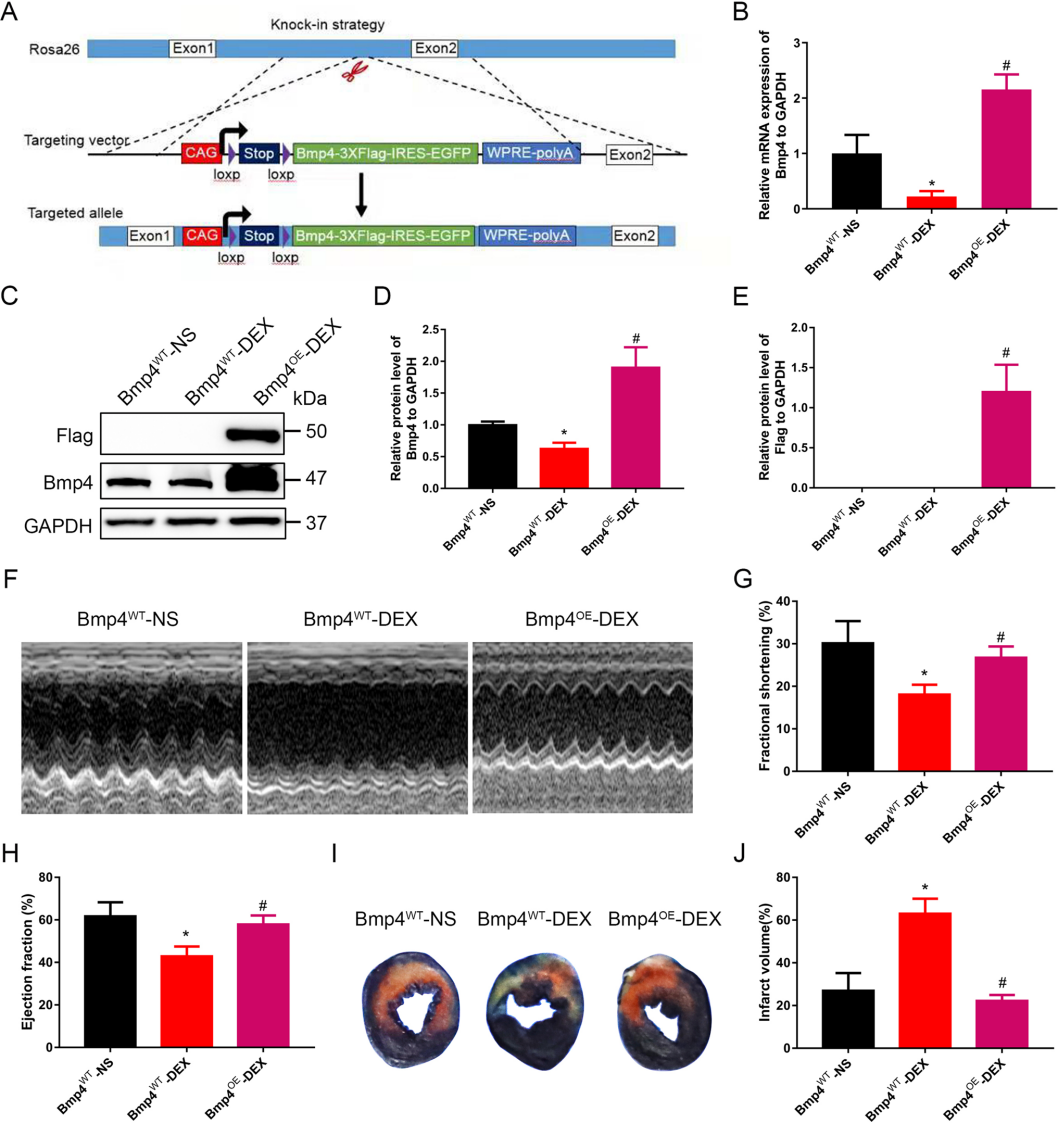
Cardiac function in male offspring of pregnant mice exposed to DEX during pregnancy is protected by BMP4 overexpression specifically in myocardium. Schematic figure showing strategy for the construction of BMP4 overexpression mice (**A**). All mice were subjected to ischemia/reperfusion (*I*/*R*, 30 min/24 h). Compared with oil-injection groups, the expression levels of *Bmp4* mRNA (**B**) and protein (**C**, **D**) in *Bmp4*^flox/flox^-αMyh6-Cre/Esr1* (*Bmp4*^OE^) mice were significantly increased after two weeks of tamoxifen injection (40 mg/kg, once a week). *n* = 6 for each group. The expression of FLAG protein linked with BMP4 was presented as well (**C**, **E**). Representative echocardiographic images of the adult male offspring in *Bmp4*^WT^ and *Bmp4*^OE^ mice prenatally exposed to NS or DEX were shown (**F**). BMP4 overexpression restored left ventricular fractional shortening (FS) and ejection fraction (EF) in *Bmp4*^OE^ mice prenatally exposed to DEX (**G**, **H**). Representative photographs of cardiac sections (**I**) and quantified data (**J**) demonstrate that BMP4 overexpression decreased infarct size in *Bmp4*^OE^ mice prenatally exposed to DEX subjected to *I*/*R* (*n* = 7–12). *p < 0.05, compared with *Bmp4*^WT^ mice prenatally exposed to NS; #*p* < 0.05, compared with *Bmp4*^WT^ mice prenatally exposed to DEX

In order to explore the role of BMP4 in myocardial protection from ischemia–reperfusion in vivo, we constructed conditional *Bmp4* overexpression (OE) mice in which BMP4 was specifically overexpressed in myocardium, with or without induction of tamoxifen (*Bmp4*^flox/flox^-αMyh6-Cre/Esr1* or *Bmp4*^flox/flox^-αMyh6-Cre) (Fig. 2A, Supplemental Fig. 1A, Supplemental Fig. 2A). Compared with oil-injection groups, the expression levels of BMP4 mRNA and protein in *Bmp4*^flox/flox^-αMyh6-Cre/Esr1* mice (Bmp4^OE^) were significantly increased after two weeks of tamoxifen treatment (Supplemental Fig. 1B, C). The FLAG protein linked to BMP4 also was present in the tamoxifen-injected group, which further confirmed the overexpression of transgenic Bmp4 (Supplemental Fig. 1C).

Consistent with the findings in rats, expression levels of both BMP4 mRNA (Fig. 2B) and protein (Fig. 2C,D) were decreased in mouse offspring prenatally treated with DEX and restored in *Bmp4*^OE^ mouse offspring. The FLAG expression was significantly evident in the myocardium of *Bmp4*^OE^ mouse offspring but not in the WT mouse offspring (Fig. 2C,E). In mice subjected to cardiac *I*/*R* injury, we found that BMP4 overexpression reversed cardiac dysfunction caused by prenatal DEX exposure, evidenced by recovered fractional shortening (FS, Fig. 2F,G) and ejection fraction (EF, Fig. 2H). Other parameters, including left ventricular internal dimensions (LVID), left ventricular anterior wall thickness (LVAW), left ventricular posterior wall thickness (LVPW), heart rate (HR), left ventricular volume (EDV, ESV), stroke volume (SV) and cardiac output (CO) in mice, are shown in Supplemental Table 2. LVID, LVAW, LVPW and ESV were notably restored in Bmp4^OE^ mice prenatally exposed to DEX.

TTC staining was used to assess the infarction area in hearts of mice in each group. It was shown that after *I*/*R*, the percentage of cardiac infarction area in mouse offspring prenatally exposed to DEX was significantly increased compared with those prenatally exposed to NS (27.11 ± 8.106% vs. 63.17 ± 6.857%) (Fig. 2I,J). The induced expression of BMP4 in myocardium significantly decreased the percentage of cardiac infarction area in the mice offspring prenatally exposed to DEX by more than 40.81% (63.17 ± 6.857% vs. 22.36 ± 2.593%) (Fig. 2I,J).

Taken together, these data suggest that BMP4 exerted an in vivo protective effect on the cardiac function from *I*/*R* injury in prenatally GC-exposed mice offspring.

### Cardiac protection by BMP4 is mediated by the intracellular Smad signaling pathway

**Fig. 3:**
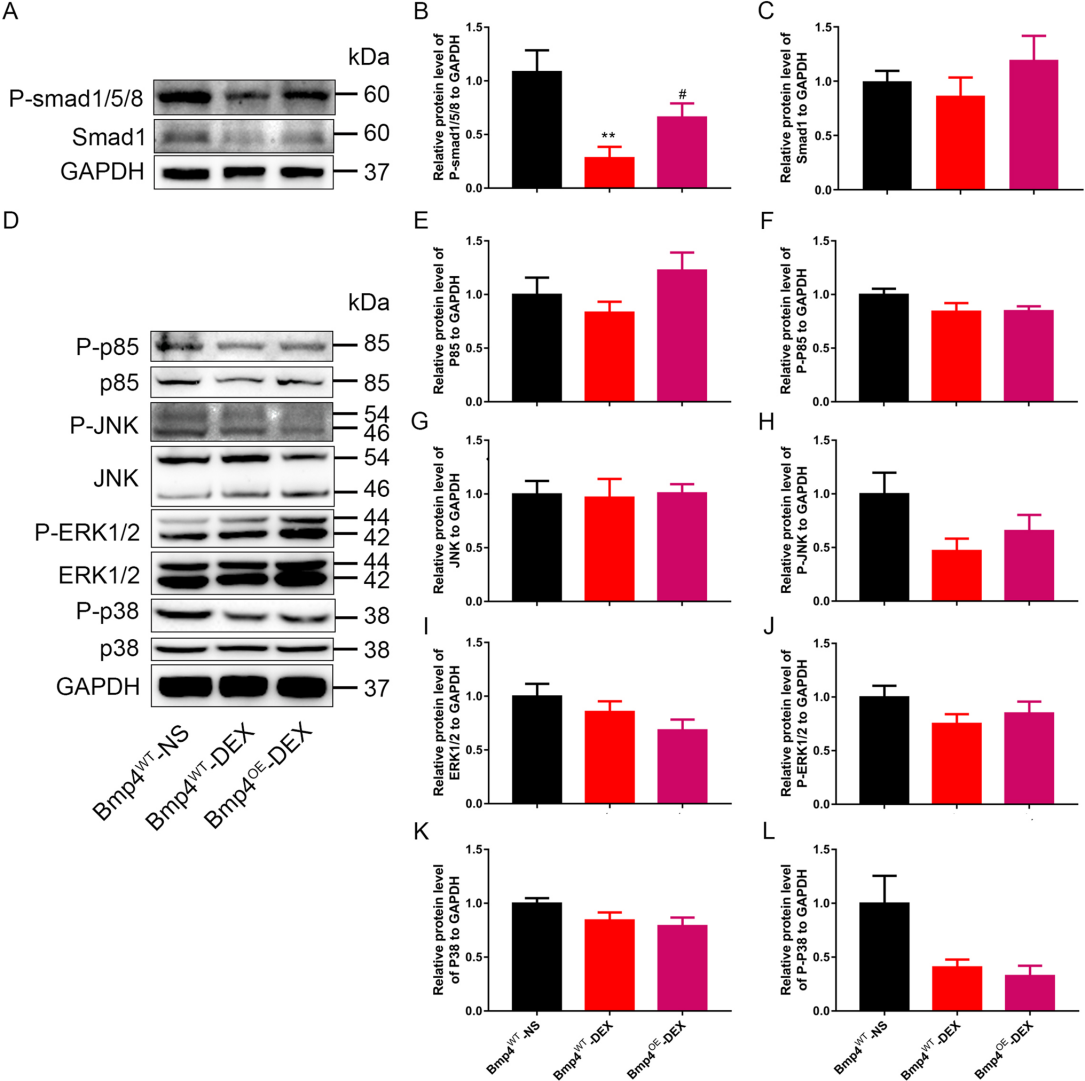
BMP4 exerts its cardiac protective effects via the intracellular Smad signaling pathway. The phosphorylation level of Smad1/5/8 (p-Smad1/5/8) was reduced remarkably in the myocardium from male mice offspring prenatally exposed to DEX compared with NS control group, which was restored in *Bmp4*^OE^ mice (**A**, **B**). The expression level of Smad1 was comparable among these groups (**A**, **C**). The protein levels of non-Smad pathway, i.e., P-85, JNK, ERK1/2 and p38 manifested no significant differences either in the WT offspring or in the *Bmp4*^OE^ offspring prenatally exposed to DEX (**D**–**L**). *n* = 6, **p < 0.01, compared with *Bmp4*^WT^ mice prenatally exposed to NS; #*p* < 0.05, compared with *Bmp4*^WT^ mice prenatally exposed to DEX

Both Smad and non-Smad signaling pathways are involved in the regulatory effects of Bmp4 on the target genes. So we next investigated which pathway BMP4 uses to exert its protective effects on cardiac function. It was shown that the phosphorylation level of Smad1/5/8 (p-Smad1/5/8) was reduced remarkably in the myocardium from male mice offspring prenatally exposed to DEX compared with NS control group (Fig. 3A,B). Intriguingly, decreased p-Smad1/5/8 was restored in *Bmp4*^OE^ mice prenatally exposed to DEX (Fig. 3A,B). The expression level of Smad1 was comparable among these groups (Fig. 3A,C). By contrast, the protein levels of non-Smad pathway signaling components, i.e., P85 (Fig. 3D–F), JNK (Fig. 3D,G,H), ERK1/2 (Fig. 3D,I,J) and p38 (Fig. 3D,K,L) manifested no significant differences either in the WT offspring or in the *Bmp4*^OE^ offspring prenatally exposed to DEX. These results collectively suggested that endogenously overexpressed BMP4 activates the Smad1/5/8 signaling pathway, which subsequently leads to the protection from cardiac dysfunction caused by excess DEX exposure during pregnancy.

### BMP4 overexpression in vivo protects cardiomyocytes from apoptosis induced by prenatal DEX exposure in male offspring mice

**Fig. 4:**
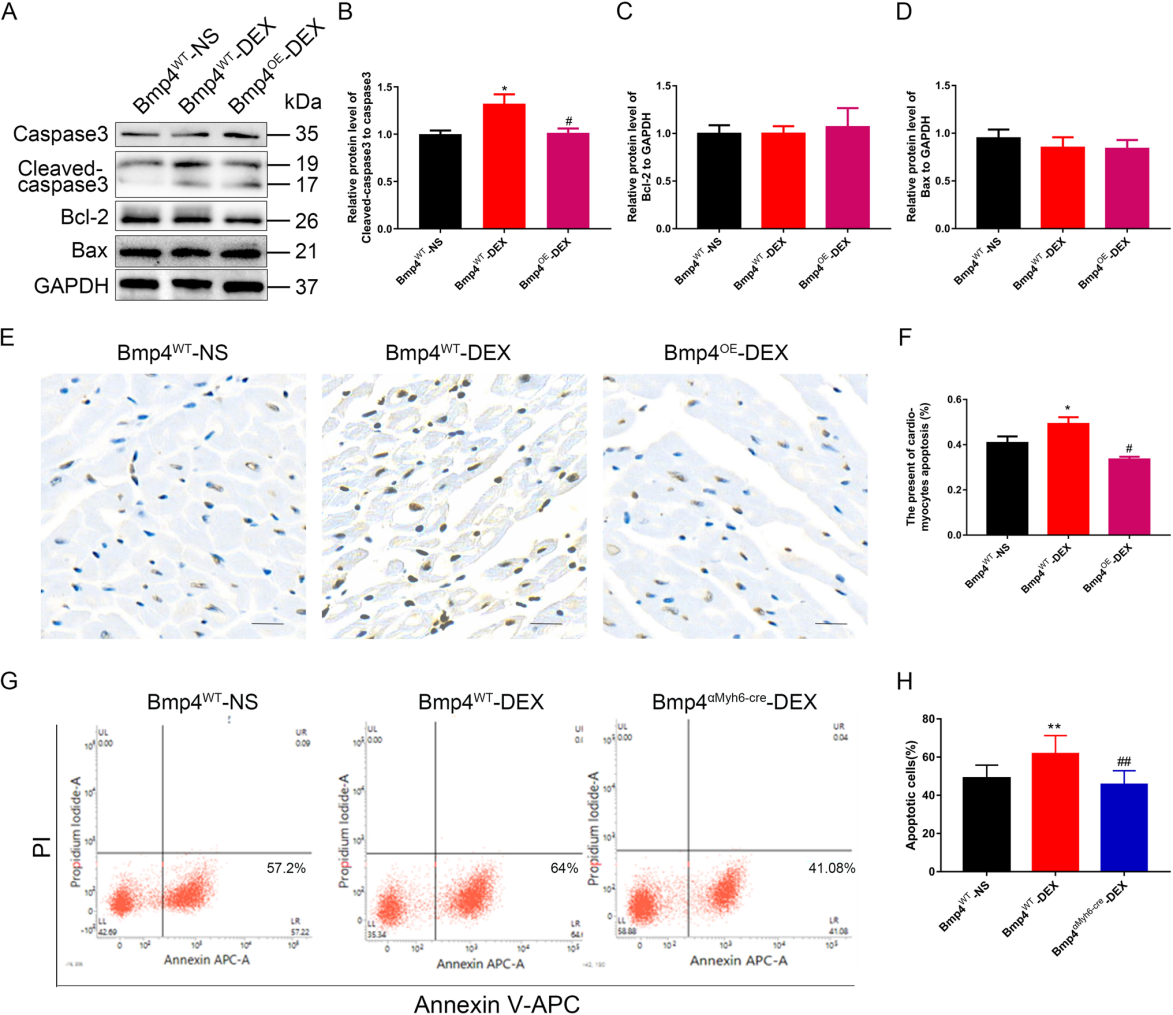
BMP4 overexpression protects against myocardial apoptosis upon *I*/*R* in male offspring mice prenatally exposed to DEX. Representative immunoblot showing the expression of cleaved-caspase 3, Bax and Bcl-2 in myocardium of male offspring prenatally exposed to NS or DEX (**A**). Quantitative analysis of cleaved-caspase 3, Bax and Bcl-2 expression is shown in panels B, C and D, respectively. *n* = 6, *p < 0.05, compared with *Bmp4*^WT^ mice prenatally exposed to NS; #*p* < 0.05, compared with *Bmp4*^WT^ mice prenatally exposed to DEX. Tunel staining showed that the percentage of apoptotic cardiomyocytes was decreased in the *Bmp4*^OE^ mice, compared with those in *Bmp4*^WT^ prenatally exposed to DEX (**E**, **F**). Original magnification: × 200; scale bar: 50 μm. *n* = 12, *p < 0.05, compared with *Bmp4*^WT^ mice prenatally exposed to NS; #*p* < 0.05, compared with *Bmp4*^WT^ mice prenatally exposed to DEX. The similar apoptotic phenotype of cardiomyocytes was observed by Flow Cytometry analysis (**G**, **H**). *n* = 6–9, **p < 0.01, compared with *Bmp4*^WT^ mice prenatally exposed to NS; ##*p* < 0.01, compared with *Bmp4*^WT^ mice prenatally exposed to DEX

Our previous work showed that DEX exposure increased cardiomyocyte apoptosis caused by *I*/*R* injury, which could be reversed by pretreatment of BMP4 in cultured rat cardiomyocytes [21]. To further investigate the effect of BMP4 on cardiomyocyte apoptosis in vivo, we detected the apoptosis in myocardium after *I*/*R* in both WT and *Bmp4*^OE^ mice prenatally exposed to DEX. We found that the cleaved-caspase 3 was decreased from myocardium in which BMP4 was specifically overexpressed, compared with that from WT offspring, both of which were prenatally exposed to DEX (Fig. 4A,B). Meanwhile, the expression of Bcl-2 and Bax showed no differences among the three groups (Fig. 4A,C,D). Moreover, the increased number of apoptotic cardiomyocytes in WT offspring prenatally exposed to DEX was significantly reduced in *Bmp4*^OE^ mice offspring prenatally exposed to DEX (Fig. 4E,F). To further verify the anti-apoptotic effect of BMP4 in cardiomyocytes in vitro, we employed another mouse model in which BMP4 was constitutively overexpressed in myocardium, without need of tamoxifen induction (*Bmp4*^αMyh6-Cre^) (Supplemental Fig. 2A). The constitutive overexpression of BMP4 in *Bmp4*^αMyh6-Cre^ transgenic mice was validated by qRT-PCR, Western Blot and fluorescence of EGFP in neonatal NMCMs (Supplemental Fig. 2B–D). We isolated and cultured neonatal mouse cardiomyocytes (NMCMs) from WT neonatal mice either exposed to NS or DEX, and *Bmp4*^αMyh6-Cre^ neonatal mice exposed to DEX. NMCMs were double stained with Annexin V and PI after *H*/*R* treatment and were analyzed by Flow Cytometry. The results showed that the apoptotic rate of cardiomyocytes in WT offspring prenatally exposed to DEX was significantly higher than that of those prenatally exposed to NS (Fig. 4G,H). However, the apoptosis of cardiomyocytes was significantly reversed in the Bmp4^αMyh6-Cre^ mice offspring prenatally exposed to DEX (Fig. 4G,H).

### BMP4 overexpression rescues mitochondrial damage and reverses oxidative stress by limiting the aberrant increase in PGC-1α in myocardium of offspring prenatally exposed to DEX

**Fig. 5:**
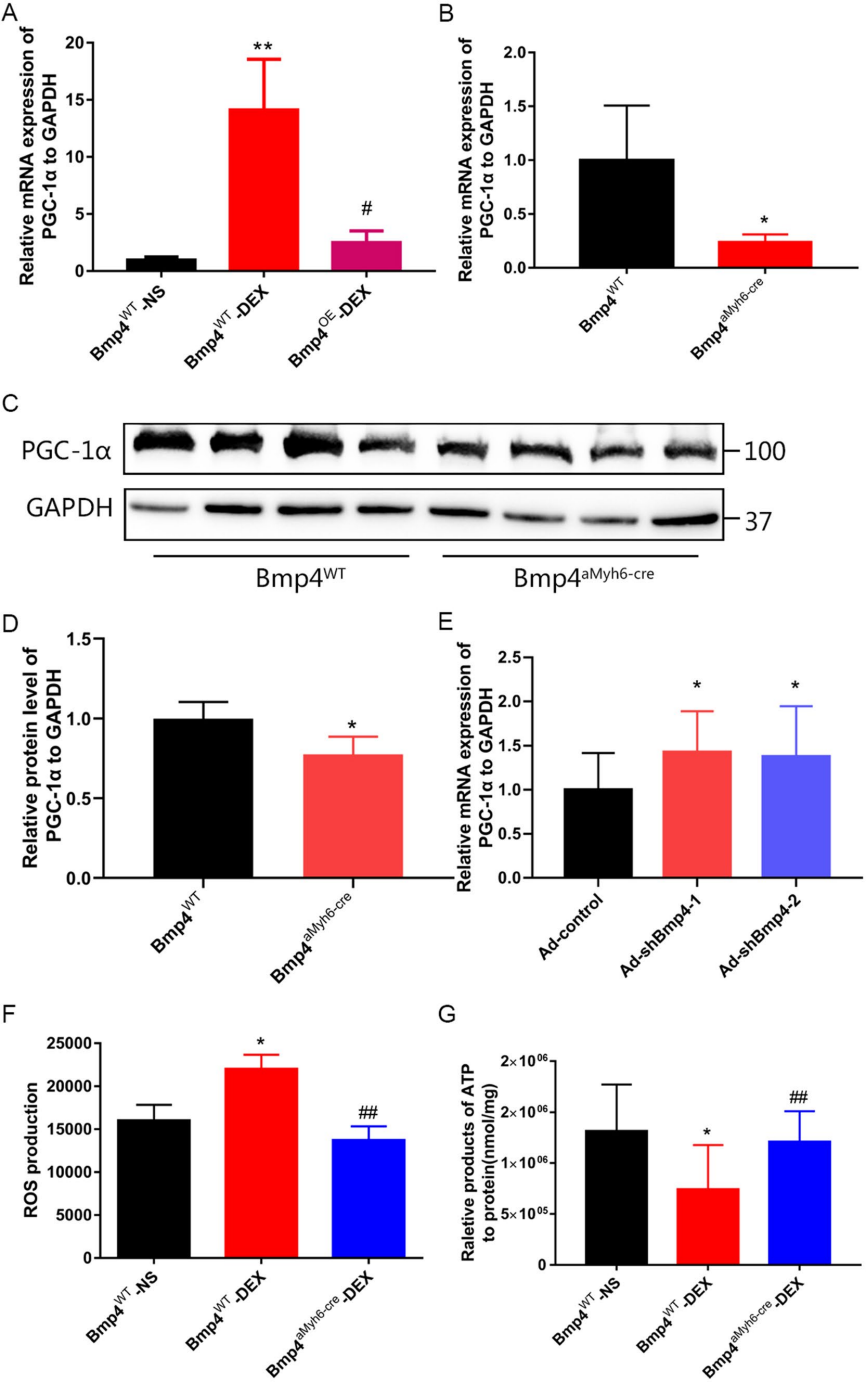
In vivo BMP4 overexpression rescues mitochondrial damage and reverses oxidative stress caused by prenatal exposure of DEX by limiting the aberrant increase in PGC-1α in offspring myocardium. PGC-1α mRNA expression was increased in cardiomyocytes from offspring prenatally exposed to DEX. This could be prevented by BMP4 overexpression in Bmp4^OE^ mice (**A**), *n* = 4–6. **p < 0.01, compared with *Bmp4*^WT^ mice prenatally exposed to NS; #*p* < 0.05, compared with *Bmp4*^WT^ mice prenatally exposed to DEX. Both the mRNA and protein levels of PGC-1α were reduced by constitutive BMP4 overexpression in *Bmp4*^flox/flox^-αMyh6-Cre (*Bmp4*^αMyh6-Cre^) mice as well (**B**–**D**). *n* = 4, *p < 0.05, compared with *Bmp4*^WT^ mice. PGC-1α mRNA levels could be upregulated by knocking down *Bmp4* in NMCMs of *Bmp4*^αMyh6-Cre^ (**E**). *n* = 7, *p < 0.05, compared with NMCMs infected with control adenovirus. The increased ROS levels and decreased ATP levels in offspring cardiomyocytes upon *I*/*R* caused by prenatal DEX exposure could both be reversed by in vivo overexpression of cardiac BMP4 in *Bmp4*^αMyh6-Cre^ mice, to a level comparable with that in WT mice prenatally exposed to NS (**F**, **G**). *n* = 6, *p < 0.05, compared with *Bmp4*^WT^ mice prenatally exposed to NS; ##*p* < 0.01, compared with *Bmp4*^WT^ mice prenatally exposed to DEX

It has been reported that peroxisome proliferator-activated receptor-γ (PPAR-γ) coactivator-1α (PGC-1α) can be upregulated by DEX in primary fetal cardiomyocytes and act as a key mediator of GC-induced maturation of fetal cardiomyocytes [30]. However, overexpression of PGC-1α has also been shown to induce mitochondrial proliferation and promote the production of reactive oxygen species (ROS) in cardiomyocytes [31–33]. This has been shown to underlie the pathogenesis of myocardial dysfunction in rats prenatally exposed to DEX [21]. Therefore, we next investigated if BMP4 could exert its cardiac protective effects by downregulating the expression of PGC-1α in prenatally DEX-exposed mice model. As expected, we found that the PGC-1α mRNA expression was increased in the cardiomyocytes from prenatally DEX-exposed mice, which was reversed by the in vivo overexpression of BMP4 in cardiomyocytes (Fig. 5A). Interestingly, both mRNA (Fig. 5B) and protein levels (Fig. 5C,D) of PGC-1α decreased in the Bmp4^αMyh6-Cre^ mice, whereas the mRNA expression of PGC-1α could be restored by knocking down BMP4 expression in cultured NMCMs infected with shBmp4 adenovirus (Supplemental Fig. 3, Fig. 5E).

In primary cultured NMCMs isolated from WT and Bmp4^αMyh6-Cre^ mice, we found that the increased ROS level and decreased ATP level in offspring cause by prenatal DEX exposure could be both reversed by in vivo overexpression of cardiac BMP4 in Bmp4^αMyh6-Cre^ mice, to a level comparable with that in WT mice prenatally exposed to NS (Fig. 5F,G).

### BMP4 overexpression inhibited DEX-enhanced mitophagy in cardiomyocytes caused by *H*/*R*

**Fig. 6:**
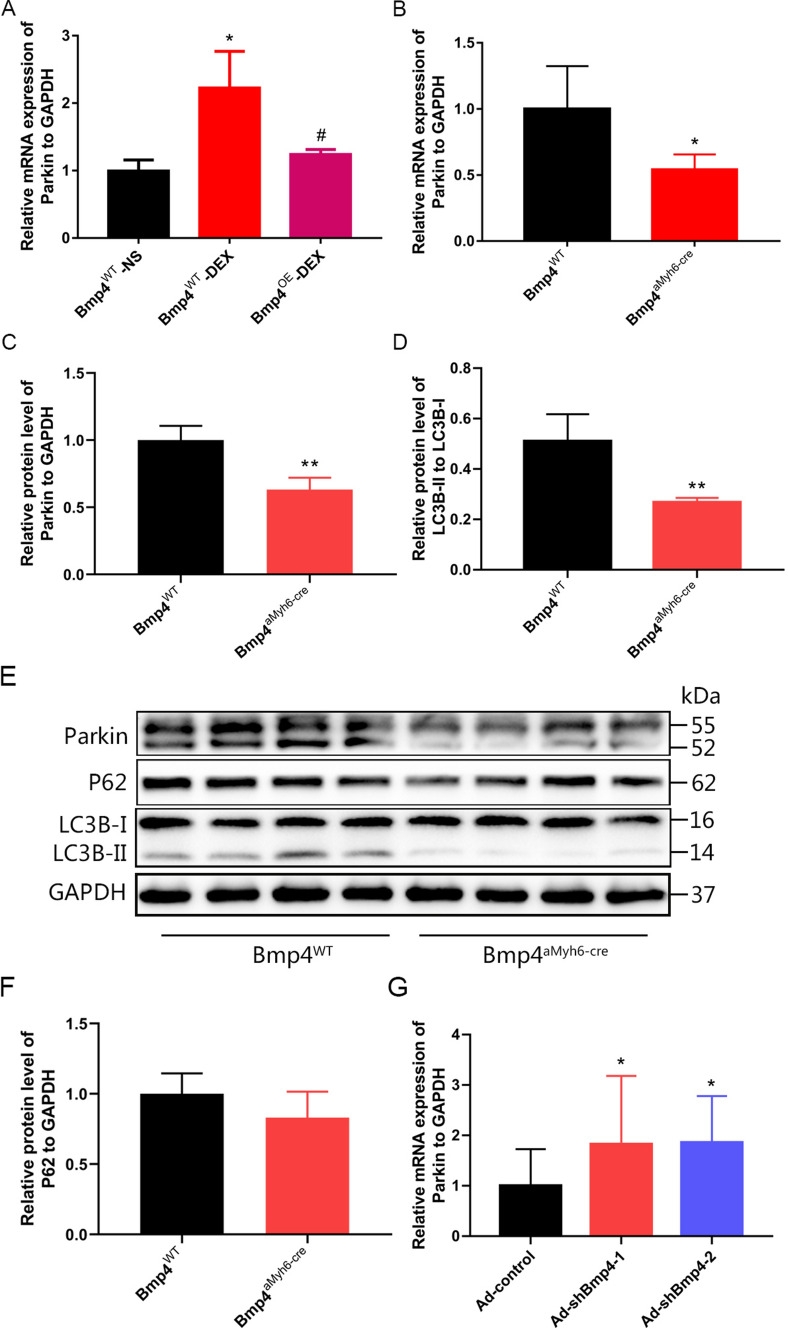
BMP4 inhibited DEX-enhanced mitophagy in cardiomyocytes caused by *H*/*R* in a Parkin-dependent manner. Parkin mRNA expression was increased in cardiomyocytes from prenatally DEX-exposed mice; this which was reversed by BMP4 overexpression in *Bmp4*^OE^ mice (**A**). *n* = 4–6. *p < 0.05, compared with *Bmp4*^WT^ mice prenatally exposed to NS; #*p* < 0.05, compared with *Bmp4*^WT^ mice prenatally exposed to DEX. Both the mRNA and protein levels of Parkin decreased in *Bmp4*^αMyh6-Cre^ mice (**B**, **C**, **E**). *n* = 4. *p < 0.05, **p < 0.01, compared with *Bmp4*^WT^ mice. The ratio between LC3II and LC3I was reduced in the *Bmp4*^αMyh6-Cre^ mice (**D**, **E**). *n* = 4, **p < 0.01, compared with *Bmp4*^WT^ mice. The protein levels of P62 manifested no differences in the myocardium between the WT and *Bmp4*^αMyh6-Cre^ mice (**E**, **F**). *n* = 4. Parkin mRNA levels also could be upregulated by knocking down *Bmp4* in NMCMs of *Bmp4*^αMyh6-Cre^ mice (**G**). *n* = 7, *p < 0.05, compared with NMCMs infected with control adenovirus

A growing number of studies suggest that accumulation of mitochondrial ROS promoted mitochondria-specific autophagy (mitophagy), and induced cell apoptosis [34]. Meanwhile, it was reported that DEX could promote Parkin-mediated mitophagy during maturation of embryonic stem cell-derived cardiomyocytes [35]. To further investigate the cellular mechanisms underlying the cardiac protective effect of BMP4 from *H*/*R* injury, we analyzed Parkin-mediated mitophagy in cardiomyocytes among different groups. It was found that the mRNA levels of Parkin were increased in cardiomyocytes from offspring prenatally exposed to DEX, compared to those of offspring prenatally exposed to NS (Fig. 6A). The upregulation of Parkin was reversed by cardiac specific overexpression of BMP4 in *Bmp4*^OE^ mice (Fig. 6A). Intriguingly, in vivo overexpression of cardiac BMP4 significantly decreased the basal mRNA (Fig. 6B) and protein expression (Fig. 6C,E) of Parkin in *Bmp4*^αMyh6-Cre^ mice. Moreover, the ratio between LC3II and LC3I was reduced in the *Bmp4*^αMyh6-Cre^ mice as well (Fig. 6D,E), while the protein levels of P62 were not different in the myocardium between the WT and Bmp4^αMyh6-Cre^ mice (Fig. 6E,F). Knocking down the BMP4 expression in cultured NMCMs by infection with shBmp4 adenovirus could restore the upregulation of Parkin (Supplemental Fig. 3, Fig. 6G). These results suggested that BMP4 could protect the cardiomyocytes from mitophagy through downregulation of Parkin.

BMP4 functions in physiological or pathological situations are generally mediated by two types of transmembrane receptors, which are type I and type II serine/threonine kinases [36]. We detected the mRNA and protein levels of BMP4 type I receptor, AVCRI, type II receptors, ACVRIIA and BMPRII, and found no significant differences in mRNA and protein expression in the myocardium among the three groups (Supplemental Fig. 4A–G). However, the inhibition of BMPRII by its specific antagonist, DMH1, significantly increased the production of ROS as well as the expression of PGC-1α and Parkin, and caused decreased production of ATP in NRCMs cells (Supplemental Fig. 4H–K). This suggests that the protective effects of BMP4 on the myocardium may be mediated by its specific receptor BMPRII.

### Pretreatment of cultured NMCMs with 5-AZA may counteract the adverse effects on cardiac function caused by DEX exposure during pregnancy

**Fig. 7:**
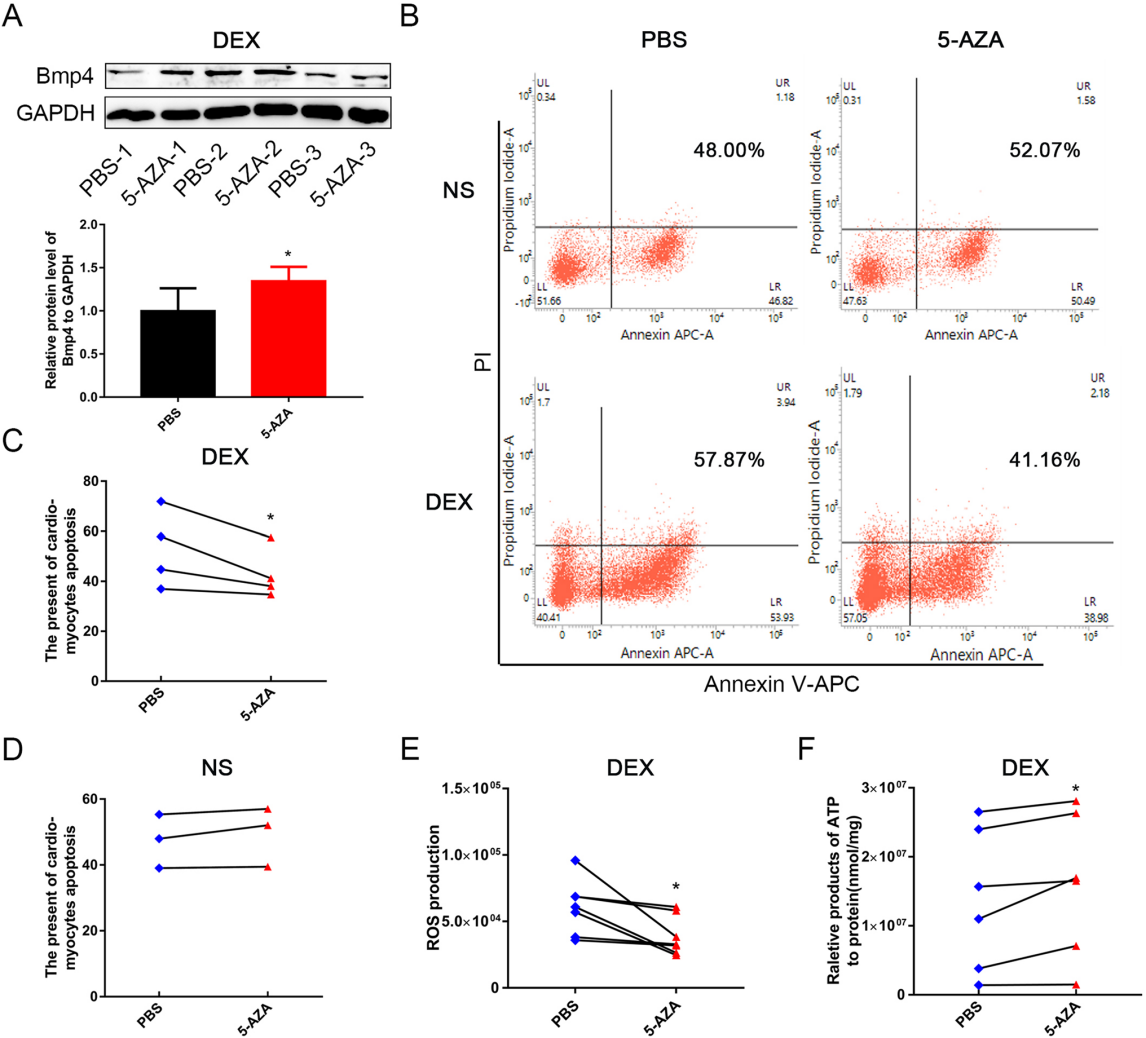
Pretreatment of cultured NMCMs with 5-AZA can counteract the adverse effects on cardiac function caused by DEX exposure during pregnancy. Pretreatment with the DNA methylation inhibitor 5-AZA increased cardiac BMP4 protein expression in WT mouse offspring that were prenatally exposed to DEX (**A**, *n* = 4). The apoptosis of cardiomyocytes was not affected by 5-AZA pretreatment in mice prenatally exposed to NS (**B**, **D**), but was significantly reduced in mice prenatally exposed to DEX after *H*/*R*, compared with the cardiomyocytes pretreated with PBS (**B**, **C**). *n* = 3–4. The accumulation of ROS was decreased (**E**, *n* = 6), while ATP levels were increased (**F**, *n* = 6) after pretreatment of 5-AZA in NMCMs isolated from myocardium of offspring of mice prenatally exposed to DEX. *p < 0.05, compared with NMCMs treated with PBS control

Finally, to assess whether blocking DNA hypermethylation induced by prenatal DEX exposure could reverse the mitochondrial damage, cell apoptosis and cardiac dysfunction upon *H*/*R*, we pretreated NMCMs isolated from WT neonatal mice prenatally exposed to DEX with 5-AZA, a DNA methylation inhibitor, before *H*/*R* treatment in vitro. As shown in Fig. 7, treatment with 5-AZA significantly increased cardiac BMP4 protein expression (Fig. 7A) and reduced the apoptosis of cardiomyocytes in mice offspring prenatally exposed to DEX compared with control group (Fig. 7B,C). However, in the NMCMs isolated from WT neonatal mice prenatally exposed to NS, pretreatment of 5-AZA did not significantly affect the apoptosis of cardiomyocytes (Fig. 7B,D). In addition, the accumulation of ROS in NMCMs isolated from WT neonatal mice prenatally exposed to DEX upon *H*/*R* treatment was decreased (Fig. 7E), while ATP levels were increased after pretreatment with 5-AZA (Fig. 7F). These results indicated that pretreatment with 5-AZA may reverse the cardiac dysfunction induced by DEX exposure during pregnancy by restoring BMP4 production and protecting mitochondrial function from overwhelmed oxidative stress in cardiomyocytes.

## Discussion

Embryonic development and early life are two major susceptibility windows during which epigenetic programming is sensitive to environmental influences, such as diet, temperature, environmental toxins, maternal behavior or childhood abuse [37]. Recently, a growing amount of evidence indicated that epigenetic mechanisms, including DNA methylation, histone modification and the actions of small non-coding RNAs, are important in developmental reprogramming. However, the precise mechanisms by which exposure to environmental factors can influence long-term outcomes are highly complex. In our previous studies, it was of great interest to determine the abnormal cardiac functions that were manifested in DEX-exposed offspring only after *I*/*R* but not in those maintained under normal oxygen conditions. Hypoxia inducible factor-1α (HIF-1α) is a key transcription factor that regulates the expression of hundreds of genes in the adaptive response to hypoxia [38]. Under normoxic conditions, HIF-1α expression is tightly regulated by ubiquitination and subsequent proteasomal degradation [39]. This regulation can be suppressed by hypoxia, which leads to elevated expression levels of active HIF-1α that translocates to the nucleus and transcriptionally regulates genes associated with physiological processes, including angiogenesis and metabolism [40]. Many studies have demonstrated that up-regulation of HIF-1α enhanced the cardioprotective effects against *I*/*R* [41–43].

In the present study, we observed that the mRNA and protein levels of Bmp4 in neonatal rat cardiomyocytes were enhanced upon *H*/*R* treatment in comparison with those cultured under normoxic condition. Intriguingly, HIF-1α is predicted by bioinformatics analysis to be a potential transcriptional factor binding to the *Bmp4* promoter, suggesting that hypoxia or ischemia-induced HIF-1α may bind to the promoter region of *Bmp4* and activate its transcription and expression to protect the myocardium from hypoxia/reoxygenation or ischemia/reperfusion injury. Our current findings using ChIP-qPCR confirmed that HIF-1α binding was indeed increased in the promoter region of *Bmp4* upon hypoxia treatment, resulting in increased *Bmp4* expression in rat H9C2 cardiomyocytes. These results were consistent with previous reports, showing that HIF-1α also upregulated transcriptional activation of *Bmp4* expression in rat pulmonary arterial smooth muscle cells and mouse embryonic stem cells [44, 45]. This further indicates that hypoxia induction of HIF-1α and HIF-1α upregulation of BMP4 is a ubiquitous mechanism among various tissues.

However, chromatin states can substantially influence the binding abilities of transcription factors to the promoters of target genes and their subsequent transactivation [46]. Our present results, from MeDIP-sequencing, Q-PCR, Western Blot and MSP, demonstrated that the promoter region of *Bmp4* in myocardium from rat male offspring exposed to GC during pregnancy is hypermethylated. Therefore, it makes perfect sense that we speculated that hypermethylation of *Bmp4* promoter impairs its binding of HIF-1α and attenuates the transactivation of Bmp4. We designed the ChIP-qPCR primers flanking the hypermethylated sequences on *Bmp4* promoter containing the HIF-1α binding site and found that HIF-1α enrichment on *Bmp4* promoter was significantly decreased after GC exposure during pregnancy, mostly due to the hypermethylation of *Bmp4* promoter. Moreover, mutation or deletion of core sequences within the hypoxia response element (HRE) of the *Bmp4* promoter that were hypermethylated caused by prenatal GC exposure prevented the induction of luciferase activity. This further indicates that hypermethylation of *Bmp4* promoter hampered its ability to bind HIF-1α, resulting in prevention of *Bmp4* transactivation and a reduced protection by BMP4 of cardiac function against *H*/*R* or *I*/*R*. Mechanistically, these results revealed that hypoxia acts as a “second hit” exaggerating the pathological changes on myocardium and explains why cardiac function of rats prenatally exposed to DEX was decreased only after ischemia–reperfusion injury, but not under normoxic conditions.

BMP4 regulates downstream signaling pathways through its receptors. Although the DNA methylation level of Bmp4 type II receptor, ACVRIIb, is also increased according to our MeDIP-sequencing results [21], neither mRNA nor protein changes of AVCRI, ACVRIIa or BMPRII were observed in the myocardium from WT or *Bmp4*^OE^ mice prenatally exposed to DEX or to NS. The abundance of ACVRIIb was too low to be detected by Western Blot. These results suggested that prenatal GC overexposure does not affect the expression of BMP4 receptors. There are generally two pathways downstream BMP4 receptor activation, namely Smad and non-Smad pathways. It was reported that BMP4 induced myocardial apoptosis by activating the JNK/MAPK pathway in *Bmp4* heterozygous KO mice during *I*/*R* [29]. However, in the present study, it was shown that BMP4 performed its cardiac protective functions via activating p-Smad1/5/8 pathway, rather than non-Smad pathways in the hearts of male offspring prenatally exposed to DEX, suggesting that the different downstream pathways activated by BMP4 may mediate different subsequent biological functions. Through activation of p-Smad1/5/8 pathway, overexpression of BMP4 exerted the anti-apoptotic effect in cardiomyocytes rather than pro-apoptotic effect mediated by non-Smad pathway.

In the present study, we found that the ROS level was increased and the ATP level was decreased in NMCMs isolated from male mouse offspring of pregnant mothers exposed to DEX. This is consistent with our previous findings in rats [21], suggesting again that mitochondrial damage and oxidative stress in cardiomyocytes caused by *H*/*R* were further negatively affected by prenatal DEX exposure. Intriguingly, these phenotypic changes could be reversed by either in vivo or in vitro overexpression of cardiac BMP4. It has been reported that PGC-1α is an essential molecule in normal mitochondrial development, function, dynamics, and in maturation of cardiomyocytes [47]. Heart-specific PGC-1α KO induces metabolic, functional, and structural abnormalities leading to dilating cardiomyopathy and heart failure [48]. However, overexpression of PGC-1α has been shown to induce mitochondrial proliferation and promote the production of ROS in cardiomyocytes [49], suggesting the fine-tuning of PGC-1α is critical for normal mitochondrial functions.

Members of the TGF-β superfamily exert different effects on the regulation of PGC-1α expression. BMP-7 significantly increased the expression of PGC-1α in a brown preadipocyte cell line [50], while the treatment of differentiated C2C12 myotubes with TGF-β suppressed PGC-1α mRNA and protein expression [51]. As similar with TGF-β, BMP4 was shown to reverse the upregulation of PGC-1α in cardiomyocytes of male offspring of DEX-treated pregnant mothers, which is consistent with the previous report that DEX can induce PGC-1α mRNA in a dose-dependent manner in primary fetal cardiomyocytes [30] and collectively suggest that DEX may induce PGC-1α by reducing BMP4 expression. In the canonical Smad-dependent pathway, phosphorylated Smad1/5/8 hetero-oligomerize with Smad4 and translocate to the nucleus, where they interact with transcription factors to regulate gene expression [52], such as suppressing Pentraxin 3 [53] and StAR [54] expression in human granulosa-lutein cells. Therefore, we speculate that the p-Smad1/5/8-Smad4 complex may suppress PGC-1α mRNA transcription and mediate the suppressive effect of BMP4 on PGC-1α. However, the specific regulatory mechanisms for these effects need to be further investigated in future studies.

Mitophagy plays an important role in cardiac *I*/*R* through Parkin-dependent and Parkin-independent processes, and dysregulation of mitophagy confers cardiac mitochondrial damage [55]. Parkin, an ubiquitin E3 ligase, adds ubiquitin to many substrates leading to interactions with LC3, a key component of autophagosomes [56]. The expression of Parkin in cardiomyocytes was found to be elevated by treatment with exogenous H_2_O_2_ or inhibited by ROS inhibitors [57]. Our present findings suggest that Parkin mRNA levels are increased in cardiomyocytes treated with *H*/*R*. This was further enhanced by prenatal DEX exposure, probably caused by increased expression of PGC-1α and ROS. However, the mRNA and protein levels of Parkin and the ratio of LC3II/LC3I were decreased in cardiomyocytes by BMP4 overexpression. Taken together, these findings strongly suggest that BMP4 may inhibit mitophagy in cardiomyocytes by blocking the PGC-1α-ROS-Parkin pathway.

## Conclusion

**Fig. 8:**
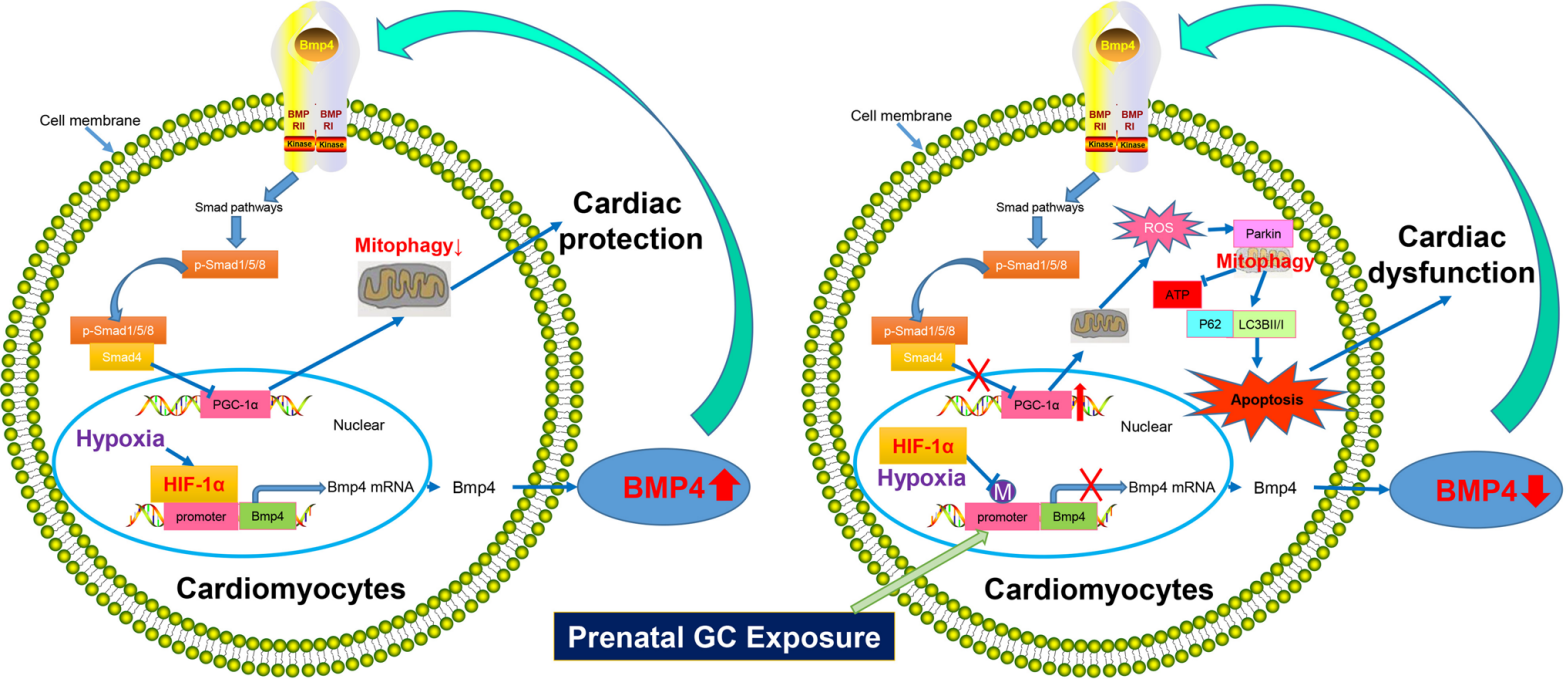
Working model. In normal cardiomyocytes, hypoxia due to ischemia–reperfusion induces the expression of transcription factor HIF-1α, which can bind to the promoter region of *Bmp4* and substantially increase BMP4 expression. BMP4 exerts cardiac protection effects by inhibiting PGC-1α expression and attenuating mitophagy of cardiomyocytes caused by hypoxia. Prenatal DEX exposure causes DNA hypermethylation of the HIF-1α binding site within the *Bmp4* promoter in cardiomyocytes, which dampens the binding activity of HIF-1α and decreases the transcription and expression of *Bmp4*. The decrease in BMP4 expression relieves its inhibitory effects on PGC-1α, which is further enhanced by DEX exposure. The elevated PGC-1α increases ROS production, leading to Parkin-dependent mitophagy. This enhances apoptosis of cardiomyocytes upon *I*/*R* or *H*/*R*, eventually leading to cardiac dysfunction in offspring of mice prenatally exposed to DEX

In summary, this study demonstrates that DNA hypermethylation of the *Bmp4* promoter in cardiomyocytes caused by prenatal DEX exposure substantially dampens promoter binding of transcription factor HIF-1α induced by cardiac ischemia and hypoxia. The resulting decrease in BMP4 expression relieves its inhibitory effects on PGC-1α, and the elevated PGC-1α increases ROS production which leads to Parkin-dependent mitophagy and enhances cardiomyocyte apoptosis upon *I*/*R* or *H*/*R*. Pretreatment with the DNA methylation inhibitor, 5-AZA, could serve as a potential therapeutic for the programming effect of GCs during pregnancy on neonatal cardiac dysfunction (Fig. 8).

## Methods and materials

### Animal model

*Bmp4*^*flox/flox*^ mice on C57BL/6 background were generated using the CRISPR-Cas9 gene editing method by the Shanghai Model Organisms (Shanghai, China). *Bmp4*^*flox/flox*^ mice were crossed with αMyh6-Cre/Esr1* (Shanghai Model Organisms) transgenic mice to generate *Bmp4*^*flox/flox*^-αMyh6-Cre/Esr1* mice. Cre recombinase (MerCreMer) induced by tamoxifen cleavage of the stop codon upstream of the *Bmp4* coding sequence, resulted in mRNA and protein overexpression of Bmp4 in juvenile and adult cardiac myocytes. In our study, tamoxifen (40 mg/kg, #T5648, Sigma-Aldrich) or corn oil (c8267, Sigma-Aldrich) was injected intraperitoneally into mice every 7 days, from the age of 8 weeks for two consecutive weeks before performing the subsequent experiments. In another set of experiments, the *Bmp4*^*flox/flox*^ mice were crossed with *αMyh6-Cre* transgenic mice (gifted by Dr. Junjie Xiao, Shanghai University) to generate *Bmp4*^*flox/flox*^*-αMyh6-Cre* mice, in which Bmp4 was constitutively overexpressed in the myocardium without the need of induction by tamoxifen.

All animal experiments were performed in accordance with the National Institutes of Health Guide for the Care and Use of Laboratory Animals, with the approval of the Committee on Ethics of Medicine, Second Military Medical University, Shanghai. Mice were housed in the SPF animal center of Second Military Medical University, under a 12-h-dark/12-h-light cycle, and allowed free access to a standard pellet chow and water. Males and females were mated at 18:00 h and separated the following morning at 06:00 h. Pregnancy was indicated by the presence of a vaginal plug, which was designated as 0.5 days post-coitus (dpc). The time to labor was documented upon delivery of the first pup or by the presence of a litter. The pregnant mice were randomly divided into DEX-treated group and NS-injected groups, which were subcutaneously injected with DEX (0.1 mg/kg/day) or the same volume of NS daily from 12.5 dpc until labor. The offspring were maintained in the same environment to adulthood (12 weeks).

### The in vivo  ischemia/reperfusion (I/R) model

After Bmp4 expression in heart was induced for 2 weeks in male mouse offspring, myocardial ischemia–reperfusion (*I*/*R*) was performed in vivo according to a previously described protocol [21]. Briefly, mice were initially anesthetized with 3% isoflurane inhalation and narcosis was maintained at 1.5–2% isoflurane, using a gas anesthesia machine (CWE Incorporated, USA). Mice were placed in a supine position, and the hair was removed from the sternal epidermis. The skin around the heart was removed, and the third and fourth ribs and intercostal muscles were punctured on the left side. The pericardium was then dissected, and the heart was squeezed out. The left coronary artery (LCA) was ligated with a 6–0 silk suture at a point 1–2 mm inferior to the left auricle. Successful ligation was confirmed when the anterior wall of the LV turned pale. The thoracotomy site was then stitched in layers with a 4–0 silk suture. After 30 min of ischemia, the silk suture was loosened and reperfusion was allowed for another 24 h.

### Primary cardiomyocyte isolation, culture, and treatment

Neonatal mouse cardiomyocytes (NMCMs) and neonatal rat cardiomyocytes (NRCMs) were isolated and cultured, as previously reported [21, 58]. The cells were cultured for 4-days before the hypoxia/reoxygenation model (*H*/*R*) was established according to the protocol described previously [21]. Briefly, cardiomyocytes cultured in serum-free and glucose-free DMEM were placed in a hypoxic chamber (HF100, Heal Force, China) containing 94% N_2_/5% CO_2_/1% O_2_ for 5 h, after which cells were subjected to reoxygenation in a standard incubator (5% CO_2_/95% air) in normal medium for 2 h.

5-Aza-2′-deoxycytidine (5-AZA, 189826, Sigma) was used as a DNA methylation inhibitor. The solution of 5-AZA was prepared with saline, and the final working concentration in the culture medium was 1 µM, as described in a previous study [59]. The control cells were cultured in the same volume of saline. After 48 h of treatment with 5-AZA or saline, NMCMs were subjected to *H*/*R*, as well.

DMH1 (T1942, Topscience, China) was used as a BMPRI antagonist and was dissolved in DMSO. The NMCMs were treated with DMH1 at a final concentration of 10 nmol/l, and the same volume of DMSO was given as the vehicle control [26].

### Echocardiography

Echocardiography of the *I*/*R* mice was performed using an ultrasound imaging system (Esaote MyLab Touch, SL3116, Italy). In short, mice were anesthetized with 2% isoflurane inhalation and scanned by two-dimensional M-mode echocardiography with a 22 MHz cardiac probe to record physiological data. Measurement techniques were consistent with the American Society of Echocardiography conventions [60]. Briefly, the probe was placed on the left side of sternum with 30-degree angle to the sternal midline, showing the left ventricular long axis section; then, the probe was rotated 90 degrees clockwise to show the left ventricular short axis section. Specifically, the left ventricular end–diastolic anterior wall thickness (AWT), posterior wall thickness (PWT), the left-ventricular end diastolic dimension (LVEDD), the left-ventricular end systolic dimension (LVESD) and heart rate were measured and recorded. Left-ventricular fractional shortening (LVFS), an index of LV systolic function, was calculated using the following equation: LVFS = (LVEDD − LVESD)/LVEDD × 100%. Left ventricular end-diastolic volume (LVEDV) = 1.04 × LVEDD^3^; left ventricular end-systolic volume (LVESV) = 1.04 × LVESD^3^. Left ventricular ejection fraction (LVEF) = (LVEDV − LVESV)/LVEDV × 100%. All ultrasound measurements were taken on an average of 5 cardiac cycles, and all investigators performing echocardiographic acquisition or analysis were blinded to treatment group.

### Quantification of I/R injury size using TTC staining

After *I*/*R*, the suture on the LCA was tightened again, and the heart was filled with 1% Evans blue (A602025, BBI, Sangon Biotech, St. Louis, China) by intravenous injection. When the skin of chest wall became blue, the heart was immediately excised and quickly frozen on dry ice. Then, the heart was cut into 2-mm-thick slices parallel to the atrioventricular groove; these were subsequently incubated with 1% 2,3,5-triphenyltetrazolium chloride (TTC, BBI, Sangon Biotech, China) in phosphate buffer (pH 7.4) at 37 °C for 3 min. Finally, the slices were immersed into 10% paraformaldehyde for 20 min. The heart sections not stained with Evans blue were identified as the risk area. The infarct area was identified as pale in color after TTC staining, since the infarct area does not contain dehydrogenase enzymes which can convert TTC into formazan and stain viable myocardium with dark red. The area at risk and the area of infarct were quantified using ImageJ (https://imagej.nih.gov/ij/). The quantification of *I*/*R* injury was expressed as a ratio of the infarct area and the area at risk.

### Methylation-specific PCR

In a previous study, we found that the methylation level of the *Bmp4* promoter region (rn5_dna range = chr15:24738861–24739100) was significantly increased in prenatally DEX-exposed rat male offspring using MeDIP sequencing. To further confirm this result, we performed methylation-specific PCR (MSP) using bisulfite-modified DNA isolated from the heart of male mouse offspring prenatally exposed to DEX. The primer pairs for the methylated (M) DNA and unmethylated (U) DNA sequences were designed using an online tool from The Li Lab (http://www.urogene.org), and the sequences of primer pairs are listed in Supplemental Table 3. Genomic DNA from the mouse hearts of the DEX or NS groups was extracted using a Mag-MK Animal Genomic DNA Extraction Kit (B518721-0100, Sangon Biotech, China). DNA bisulphite conversion was performed with a commercially available kit (EpiMark^®^ Bisulfite Conversion Kit, #E3318S, New England Biolabs). DNA Amplification was achieved in a 25 μl reaction volume containing 0.5 μl of each primer, 2 μl of modified DNA, 0.125 μl EpiMark Hot Start Taq DNA Polymerase (#M0490S, New England Biolabs). The PCR reaction conditions were optimized according to the manufacturer's instructions. The PCR products (134 bp) were visualized on a 2% agarose gel stained with 4S GelRed (Sangon Biotech, China) which can be illuminated by UV.

### RNA extraction and quantitative real-time PCR analysis

Total RNA was isolated from heart tissues or cells using TRIzol reagent (15596018, Invitrogen, America). The concentration of total RNA was determined by measuring absorbance at OD260 on a Microplate Reader. cDNA synthesis was performed using PrimeScript™ RT reagent Kit (RR047A, Takara, China), and 0.5 μg of mRNA from each sample was reverse transcribed into cDNA, according to the manufacturer’s instructions. Quantitative analysis of mRNA was performed on a CFX Connect Real-Time PCR Detection System (Bio-Rad) using SYBR Green Real-time PCR Master Mix (QPK-201, TOYOBO, Japan). The cycling conditions were 95 °C for 3 min, followed by 40 cycles at 95 °C for 15 s, 50 °C for 30 s, and 72 °C for 15 s. The primers are listed in Supplemental Table 4. As a negative control for all of reactions, distilled water was used in place of cDNA. The specificity of primers was verified by examining the melting curve, and amplification of the GAPDH was determined for sample loading and normalization. Each sample was amplified in triplicate, and the mean *C*_t_ value of each sample was calculated. The comparative *C*_t_ method (2^−ΔΔ*C*t^) was employed to compare the relative fold changes among different samples [61].

### Western Blot

Total proteins were extracted from tissues and cells using RIPA buffer (Cell Signaling). Equivalent amounts of protein determined by BCA assay were resolved by 10% Bis–Tris gel electrophoresis and blotted to Immobilon PVDF membranes (Millipore). The following primary antibodies and dilutions were used: anti-BMP4 (1:1000; ab39973, Abcam); anti-GAPDH (1:2000; sc-32233, Santa Cruz);anti-Flag (1:1000; F1804, Sigma); anti-ACVRIIA (1:1000; ab134082, Abcam); anti-ACVRI (1:1000; 4398 s, Cell Signaling); anti-BMPRII (1:1000; 6979 s, Cell Signaling); anti-p-Smad1/5/8 (1:1000; 13820 s, Cell Signaling); anti-Smad1 (1:1000; 9743 s, Cell Signaling); anti-P85 (1:1000; 4292S, Cell Signaling); anti-Phospho-SAPK/JNK (Thr183/Tyr185) (1:1000; 4668S, Cell Signaling); anti-SAPK/JNK (1:1000; 9252 s, Cell Signaling); anti-P-ERK1/2 (1:1000; 8544S, Cell Signaling); anti-ERK1/2 (1:1000; 4695S, Cell Signaling); anti-P-P38 (1:1000; 4511S, Cell Signaling); anti-P38 (1:1000; 8690S, Cell Signaling); anti-Capspase3 (1:1000; 9662S, Cell Signaling); anti-LC3B (1:1000; ab192890, Abcam); anti-Parkin (1:1000; ab77924, Abcam); anti-P62 (1:1000; ab109012, Abcam); anti-PGC1 alpha + beta (1:1000; ab188102, Abcam). The secondary antibody was horseradish peroxidase-conjugated anti-rabbit or anti-mouse IgG (Jackson). The ratio of band intensity of target protein to that of GAPDH was calculated as the relative expression level of protein.

### Transcription factor-binding site analysis

In order to identify the putative transcription factor (TF) binding sites within the methylated regions of the *Bmp4* promoter, bioinformatics analysis was performed using LASAGNA-Search 2.0 online tool (https://biogrid-lasagna.engr.uconn.edu/lasagna_search/) with TRANSFAC matrices and aligned models.

### Chromatin immunoprecipitation (ChIP)-qPCR

ChIP assays were performed using a SimpleChIP^®^ Plus Enzymatic Chromatin IP Kit (#9005, CST, USA) following the manufacturer’s instructions. Briefly, tissues (25 mg each sample) were crosslinked using formaldehyde (#12606, CST, USA) and digested into a single-cell suspension using a Tissuelyser (#12606, CST, USA). The nuclear extracts from pelleted cells were then sonicated, and the genomic DNA was fragmented to an average length of 200–1000 bp using a Bioruptor^®^Pico (Diagenode, America). Immunoprecipitation was conducted with an anti-HIF-1α antibody (Abcam) or IgG (Beyotime, China). Crosslinking was reversed by incubation in Elution buffer at 62 °C for 2 h. DNA was then extracted from samples using column purification and amplified by Real-time PCR.

### Cell line culture and transfection

Rat myocardium cell line H9C2 cells (Chinese Academy of Science, Shanghai, China) were cultured in DMEM (11995065, Gibco, USA) containing 10% (v/v) fetal bovine serum (FBS, 10099-141C, Gibco, USA) and 1% (v/v) penicillin–streptomycin solution (15140-122, Thermo, USA) at 37 °C in an incubator (HFsafe1200LC) with 5% CO_2_ and 95% air. Lipofectamine™ 3000 transfection reagent (L3000-008, Invitrogen, USA) was utilized for transfection according to the manufacturer’s instructions.

### Plasmids and luciferase assay

The methylated region of the rat *Bmp4* promoter (rn5_dna range = chr15:24738861–24739100) was amplified and cloned into the pGL4.23-basic vector (Promega). The Hieff Mut™ Site-Directed Mutagenesis Kit (11003ES10, Yeasen) was used to mutate four nucleotides (gcaCGTCt to gcaAAGTt), or delete the four nucleotides (CGTC) in the putative HIF-1α binding site within the methylated region of the *Bmp4* promoter, according to the manufacturer’s protocols. All constructs were confirmed by DNA sequencing at Sangon Biotech Inc. (Shanghai, China) using the primers listed in Supplemental Table 5.

H9C2 cells were seeded in 24-well plates and cultured for 24 h. pGL4.23-basic, pGL4.23-Bmp4 or pGL4.23-mutants were transfected into the H9C2 cells. Each plasmid was transfected in quadruplicate. pRL-TK plasmid (Renilla) was cotransfected as an endogenous control for normalization. After 24 h, the plate was removed into the tri-gas incubator (94%N_2_/5%CO_2_) for incubation in a hypoxic (1% oxygen) environment for another 24 h. The cells were then washed three times with cold phosphate-buffered saline (PBS) and lysed with luciferase assay buffer. Luciferase activity was analyzed using the dual-luciferase reporter assay system (E1960, Promega) according to the manufacturer’s instructions. Transcriptional activities were determined as the ratio of firefly luciferase activity to Renilla luciferase activity. This experiment was repeated three times.

### TUNEL staining

After *I*/*R*, the hearts were washed with PBS and fixed with paraformaldehyde. Paraffin-embedded heart sections were deparaffinized, rehydrated and incubated with proteinase K at 37 °C for 20 min to retrieve the antigens. Apoptotic death of cardiomyocytes was determined using TUNEL kit (Beyotime, Shanghai, China) according to the manufacturer’s instructions. Briefly, the cells were permeabilized with 0.1% Triton X-100 and incubated with 50 μl TUNEL reaction mixture in a humidified atmosphere for 60 min at 37 °C in the dark. To detect the nuclei, the cells were counter-stained with hematoxylin for 10 min at room temperature. The fraction of apoptotic cells was determined as the average ratio between the TUNEL-positive cells (brown staining) and the hematoxylin-positive cardiac myocyte nuclei (blue staining), from five randomly selected fields under the microscope (magnification 400 ×).

### Flow cytometry analysis

Apoptosis of primary cardiomyocytes was determined using an APC Annexin V apoptosis detection kit with PI (640932, BioLegend) according to the manufacturer’s protocol. The cultured cells were dispersed using EDTA-trypsin and resuspended in 100 μl binding buffer. After incubation with 5 μl Annexin V-APC and 7 μl PI for 15 min in the dark, another 300 µl of binding buffer were added and the stained cells were examined on a BD FACSVerse (BD Biosciences, USA) within 1 h. Fragments of dead cells and debris were gated by forward- and side-scatter analysis with the FACSSuite software (BD Biosciences).

### Determination of cellular reactive oxygen species (ROS)

The levels of ROS in cells were detected using 2′–7′-dichlorodihydrofluorescein diacetate (DCFH-DA; S0033S, Beyotime) following the manufacturer's instructions. Briefly, the cultured cells subjected to *H*/*R* treatment were washed with FBS-free culture medium and incubated with 10 μM oxidation-sensitive fluorescent probe DCFH-DA in the carbon dioxide incubator for 20 min at 37 °C. The stained cells were subjected to trypsin–EDTA digestion, and 2 × 10^4^ cells in 100 μl DMEM were placed into a 96-well plate. The relative ROS levels generated by the cells were measured by a multifunctional microplate reader (Cytation5, BioTek, USA). The absorbance values were detected at the emission wavelength of 488 nm and the excitation wavelength of 525 nm.

### Detection of cellular ATP levels

The ATP levels in cells were assessed using an Enhanced ATP Assay Kit (S0027, Beyotime, China). Briefly, cardiomyocytes in primarily-culture subjected to *H*/*R* treatment in 6-well plates were lysed in 200 μl lysis buffer on ice and collected by centrifugation. Twenty microliters of supernatant were added to a 96-well plate containing 100 μL ATP detection buffer in triplicate, and the luminescence was detected by multifunctional microplate reader (Cytation5, BioTek, USA). The concentration of ATP was calculated according to a standard curve. The protein concentration of each sample was also determined and used for normalization of the ATP levels.

### Adenovirus construction and adenoviral infection

Knockdown of Bmp4 was accomplished using specific recombinant adenovirus small hairpin RNA (shRNA). The shRNA-Bmp4 recombinant adenovirus Adeno-U6-shRNA-CMV-mCherry (Ad-shBmp4-1 and Ad-shBmp4-2) and the empty control recombinant adenovirus (Ad-shNC) were purchased from Obio Technology Company (Shanghai, China). The target sequences of shRNA were as follows: shBmp4-1 (5′-GGATTACATGAGGGATCTTTA-3′), shBmp4-2 (5′-CCCTAGTCAACTCTGTTAATT-3′), and shNC (5′-TTCTCCGAACGTGTCACGT-3′). Adenoviral infection was performed according to the manufacturer’s instructions. Briefly, the NMCMs were cultured in serum-free and glucose-free DMEM for 48 h, before the cells were infected with recombinant adenovirus at a multiplicity of infection of 10 for 6 h. Then, the medium was removed, and the infected cells were allowed to recover in normal medium for another 24 h. Finally, the cells were harvested for the subsequent analysis.

### Statistics

Statistical analysis was performed using GraphPad Prism 7 software, and all data are presented as mean ± SEM, or as the mean percentage of control ± SEM, in some cases. Differences between two groups were assessed by Student’s *t* test. Differences between multiple groups were analyzed by one-way ANOVA followed by a multiple comparisons test. *p* < 0.05 indicated a statistically significant difference.

## Supplementary Information

Below is the link to the electronic supplementary material.

Supplementary file1 (DOCX 629 KB)

## Data Availability

The datasets used and/or analyzed during the current study are available from the corresponding author on reasonable request.
