# Supplementary material for: Hypermethylation of the Bmp4 promoter dampens binding of HIF-1α and impairs its cardiac protective effects from oxidative stress in prenatally GC-exposed offspring
Source: Cell Mol Life Sci. 2023 Feb 6;80(3):58. doi: 10.1007/s00018-023-04703-0 (PMC9902418; doi:10.1007/s00018-023-04703-0)
Supplement: Supplementary file 1 — Supplementary file1 (DOCX 629 KB) [file 18_2023_4703_MOESM1_ESM.docx]

**Supplemental Tables**

**Supplemental Table 1.** The putative transcription factors (TFs) binding sites at the methylated regions of the *Bmp4* promoter,

| Name | Sequence | Position | Strand | Score | p-value | E-value |
| --- | --- | --- | --- | --- | --- | --- |
|  |  | (0-based) |  |  |  |  |
| [ZNF354C](https://biogrid-lasagna.engr.uconn.edu/lasagna_search/jaspar_core_matrices/MA0130.1.html) | GTCCAG | 3 | + | 8.43 | 0.0002 | 0.047 |
| [(MA0130.1)](https://biogrid-lasagna.engr.uconn.edu/lasagna_search/jaspar_core_matrices/MA0130.1.html) |  |  |  |  |  |  |
| [MZF1_1-4](https://biogrid-lasagna.engr.uconn.edu/lasagna_search/jaspar_core_matrices/MA0056.1.html) | CGGGGA | 201 | + | 6.51 | 0 | 0 |
| [(MA0056.1)](https://biogrid-lasagna.engr.uconn.edu/lasagna_search/jaspar_core_matrices/MA0056.1.html) |  |  |  |  |  |  |
| [MZF1_1-4](https://biogrid-lasagna.engr.uconn.edu/lasagna_search/jaspar_core_matrices/MA0056.1.html) | CGGGGA | 69 | - | 6.51 | 0 | 0 |
| [(MA0056.1)](https://biogrid-lasagna.engr.uconn.edu/lasagna_search/jaspar_core_matrices/MA0056.1.html) |  |  |  |  |  |  |
| [Nr2e3](https://biogrid-lasagna.engr.uconn.edu/lasagna_search/jaspar_core_matrices/MA0164.1.html) | CAACCTT | 218 | - | 7.71 | 0.00085 | 0.199 |
| [(MA0164.1)](https://biogrid-lasagna.engr.uconn.edu/lasagna_search/jaspar_core_matrices/MA0164.1.html) |  |  |  |  |  |  |
| [HIF1A::ARNT](https://biogrid-lasagna.engr.uconn.edu/lasagna_search/jaspar_core_matrices/MA0259.1.html) | AGACGTGC | 155 | - | 10.55 | 5.00E-05 | 0.0117 |
| [(MA0259.1)](https://biogrid-lasagna.engr.uconn.edu/lasagna_search/jaspar_core_matrices/MA0259.1.html) |  |  |  |  |  |  |
| [Pax2](https://biogrid-lasagna.engr.uconn.edu/lasagna_search/jaspar_core_matrices/MA0067.1.html) | AGTCACTG | 130 | + | 9.06 | 0.00015 | 0.035 |
| [(MA0067.1)](https://biogrid-lasagna.engr.uconn.edu/lasagna_search/jaspar_core_matrices/MA0067.1.html) |  |  |  |  |  |  |
| [ELK1](https://biogrid-lasagna.engr.uconn.edu/lasagna_search/jaspar_core_matrices/MA0028.1.html) | TTATCTGAAG | 211 | + | 8.99 | 0.000425 | 0.098 |
| [(MA0028.1)](https://biogrid-lasagna.engr.uconn.edu/lasagna_search/jaspar_core_matrices/MA0028.1.html) |  |  |  |  |  |  |
| [SP1](https://biogrid-lasagna.engr.uconn.edu/lasagna_search/jaspar_core_matrices/MA0079.1.html) | CAGGCGGGGA | 69 | - | 6.83 | 0.000975 | 0.225 |
| [(MA0079.1)](https://biogrid-lasagna.engr.uconn.edu/lasagna_search/jaspar_core_matrices/MA0079.1.html) |  |  |  |  |  |  |
| [MIZF](https://biogrid-lasagna.engr.uconn.edu/lasagna_search/jaspar_core_matrices/MA0131.1.html) | CAACCTCCGC | 25 | + | 7.93 | 0.000775 | 0.179 |
| [(MA0131.1)](https://biogrid-lasagna.engr.uconn.edu/lasagna_search/jaspar_core_matrices/MA0131.1.html) |  |  |  |  |  |  |

**Supplemental Table 2.** The parameters indicating cardiac functions upon I/R assessed in Bmp4^flox/flox^-αMyh6-Cre/Esr1* mice and WT mice prenatally exposed to NS or DEX.

|  | Bmp4^WT^-NS | Bmp4^WT^-DEX | *p*-Value | Bmp4^OE^-DEX | *p*-Value |
| --- | --- | --- | --- | --- | --- |
| n | 7 | 9 |  | 12 |  |
| LVIDd(mm) | 3.15±0.1362 | 3.35±0.1476 | 0.4117 | 2.975±0.1173 | 0.1791 |
| LVIDs(mm) | 2.15±0.2187 | 2.75±0.1803 | 0.0808 | 2.275±0.1359^#^ | 0.0415 |
| LVAWd(mm) | 0.67±0.03417 | 0.605±0.02032 | 0.1599 | 0.67±0.03249 | 0.0981 |
| LVAWs(mm) | 1.15±0.05491 | 0.85±0.02863*** | 0.0002 | 1.05±0.04544^##^ | 0.0021 |
| LVPWd(mm) | 0.61±0.03836 | 0.545±0.03551 | 0.3224 | 0.625±0.03276 | 0.241 |
| LVPWs(mm) | 0.94±0.0748 | 0.82±0.04567 | 0.0925 | 1.01±0.05303^#^ | 0.0431 |
| HR(min^-1^) | 514±27.37 | 514±15.05 | 0.7954 | 514±19.33 | 0.6501 |
| EDV(ml) | 0.08059±0.01004 | 0.09622±0.01153 | 0.3555 | 0.06844±0.0087 | 0.1647 |
| ESV(ml) | 0.0266±0.008637 | 0.05442±0.01092 | 0.1011 | 0.03138±0.005703^#^ | 0.0415 |
| SV(ml) | 0.04699±0.005912 | 0.03587±0.004028 | 0.1525 | 0.04263±0.004522 | 0.4695 |
| CO(ml) | 26.34±3.708 | 19.48±1.925 | 0.1861 | 19.64±2.449 | 0.5651 |

Values were expressed as mean ± SEM. ****p<0.001*, compared with Bmp4^WT^ mice prenatally exposed to NS; #p<0.05, ##p<0.01, compared with Bmp4^WT^ mice prenatally exposed to DEX.

**Supplemental** **Table 3**. List of custom designed primers for MSP.

| Primer name | Sequence |
| --- | --- |
| R-Q-bmp4-M-F | GTTCGTAGTTGGAGTTTTAGTCGT |
| R-Q-bmp4-M-R | AAATAAAAACGTACCCCACGTC |
| R-Q-bmp4-U-F | GTTTGTAGTTGGAGTTTTAGTTGT |
| R-Q-bmp4-U-R | AAATAAAAACATACCCCACATC |

**Supplemental** **Table 4**. List of custom designed primers for real-time RT-PCR.

| Primer name | Sequence |
| --- | --- |
| Q-m-Bmp4-F | ATCGTTACCTCAAGGGAGTGG |
| Q-m-Bmp4-R | ATGGCATGGTTGGTTGAGTT |
| Q-m-BMPRII-F | GAGAACTTTCCACCCCCT |
| Q-m-BMPRII-R | ACTGCTCCGTATCGACCC |
| Q-m-ACVRIIA-F | TGGGAAAGAGACAGAACC |
| Q-m-ACVRIIA-R | CCCTCACAGCAACAAAAG |
| Q-m-ACVRIA-F | TCCCGAGACGAGAAGTCA |
| Q-m-ACVRIA-R | AATCCGAAGGCAGCTAAC |
| Q-m-PGC1a-F | GAACAAGACTATTGAGCGAACC |
| Q-m-PGC1a-R | GAGTGGCTGCCTTGGGTA |
| Q-m-Parkin-F | AGCAGTTAAACCCACCTACA |
| Q-m-Parkin-R | AGACATCGTCCCAGCAAG |
| Q-m-GAPDH-F | CCTTCCGTGTTCCTACCC |
| Q-m-GAPDH-R | AAGTCGCAGGAGACAACC |
| Q-R-Bmp4-F | ATCGTTACCTCAAGGGAGTGG |
| Q-R-Bmp4-R | ATGGCATGATTGGTTGAGTT |
| Q-R-GAPDH-F | ATCACTGCCACTCAGAAGA |
| Q-R-GAPDH-R | ACATTGGGGGTAGGAACAC |

**Supplemental** **Table 5**. List of custom designed primers for vector construction.

| Primer name | Sequence |
| --- | --- |
| R-O-bmp4-F | GGGGGTACCTGGGTCCAGCCCCTGCGA |
| R-O-bmp4-R | CCGCTCGAGTGCACCCTGATCCCTCA |
| R-O-bmp4-mut-F | GCACGCAGGGGACGTGGGGCAAAGTTCTACCTGCGGCGCGCAGTGC |
| R-O-bmp4-mut-R | GCACTGCGCGCCGCAGGTAGAACTTTGCCCCACGTCCCCTGCGTGC |
| R-O-bmp4-del-F | GCACGCAGGGGACGTGGGGCATCTACCTGCGGCGCGCAGTGC |
| R-O-bmp4-del-R | GCACTGCGCGCCGCAGGTAGATGCCCCACGTCCCCTGCGTGC |

**Supplemental Figures**

**
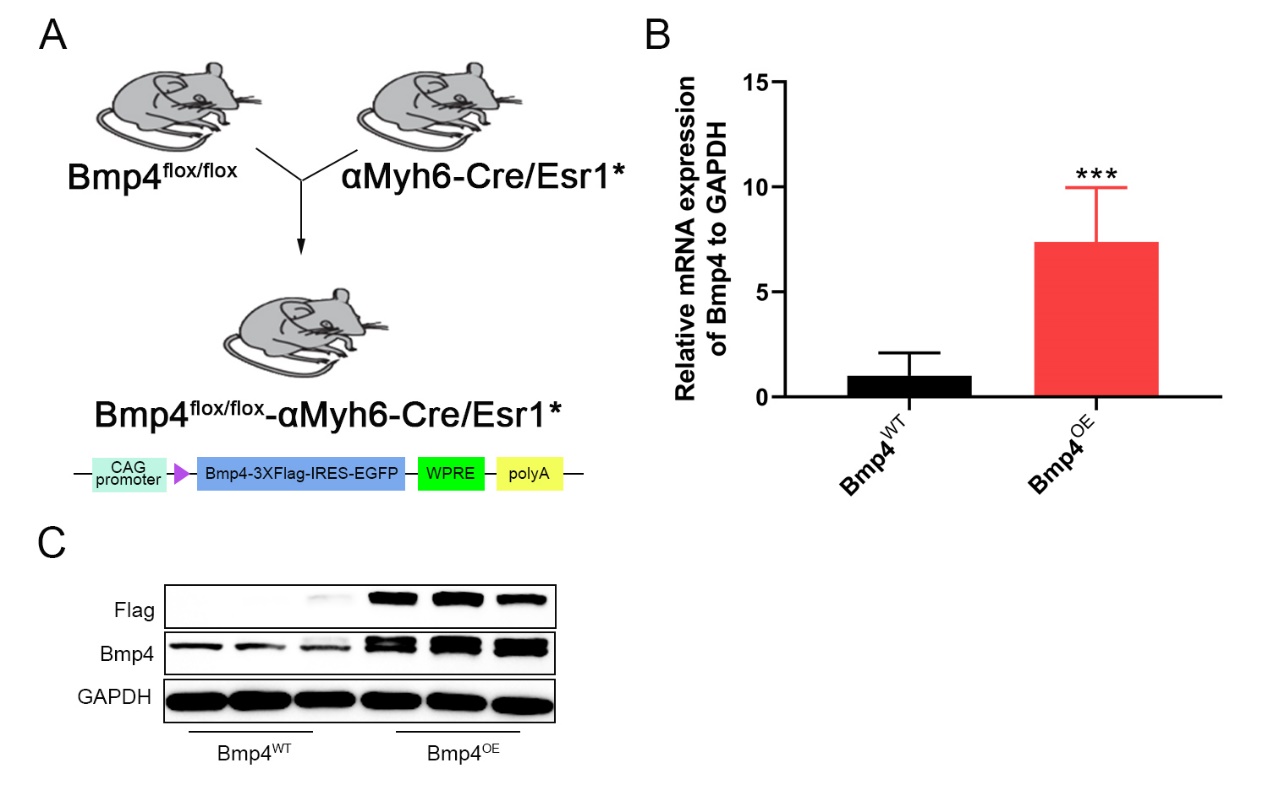
**

sFig.1: The generation of *Bmp4*^flox/flox^ -αMyh6-Cre/Esr1* mice. Schematic diagram of *Bmp4*^flox/flox^-αMyh6-Cre/Esr1* mouse construction (A). The mRNA and protein expression of *Bmp4* induced by tamoxifen in myocardium from *Bmp4*^flox/flox^-αMyh6-Cre/Esr1* mice (B and C). n=3, ***p<0.001, compared with *Bmp4*^WT^ mice.


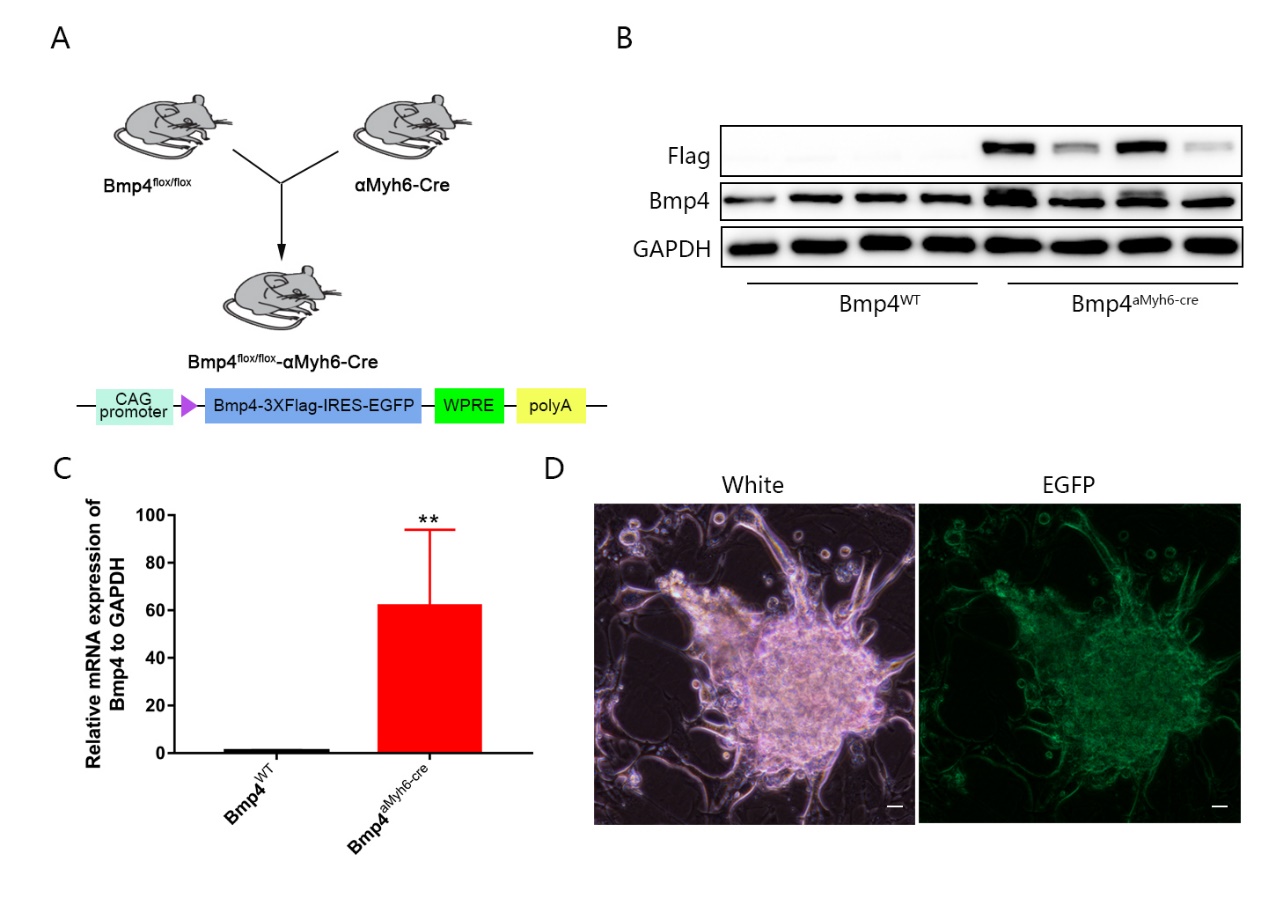


sFig.2: The generation of *Bmp4*^flox/flox^-αMyh6-Cre mice. Schematic diagram of *Bmp4*^flox/flox^-αMyh6-Cre mouse construction (A). The protein and mRNA expression of *Bmp4* in NMCMs from *Bmp4*^flox/flox^-αMyh6-Cre mice (B and C). n=4, **p<0.01 compared with *Bmp4*^WT^ mice. The EGFP fluorescence expressed in *Bmp4*^flox/flox^-αMyh6-Cre neonatal cardiomyocyte was shown (D).


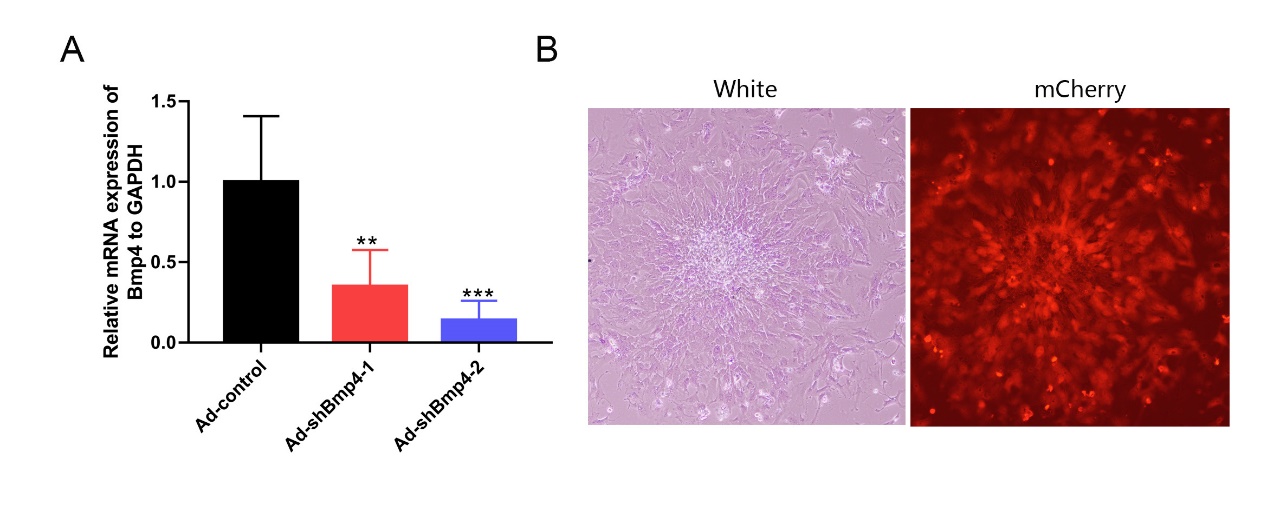


sFig.3: The mRNA expression of *Bmp4* was knocked-down by specific small hairpin RNA (shRNA) targeting Bmp4 through infection of recombinant adenovirus (A). The infection efficiency of recombinant adenovirus was shown by the mCherry fluorescence in NMCMs from *Bmp4*^flox/flox^-αMyh6-Cre mice (B). n=6, **p<0.01, ***p<0.001 compared with NMCMs infected with control adenovirus.


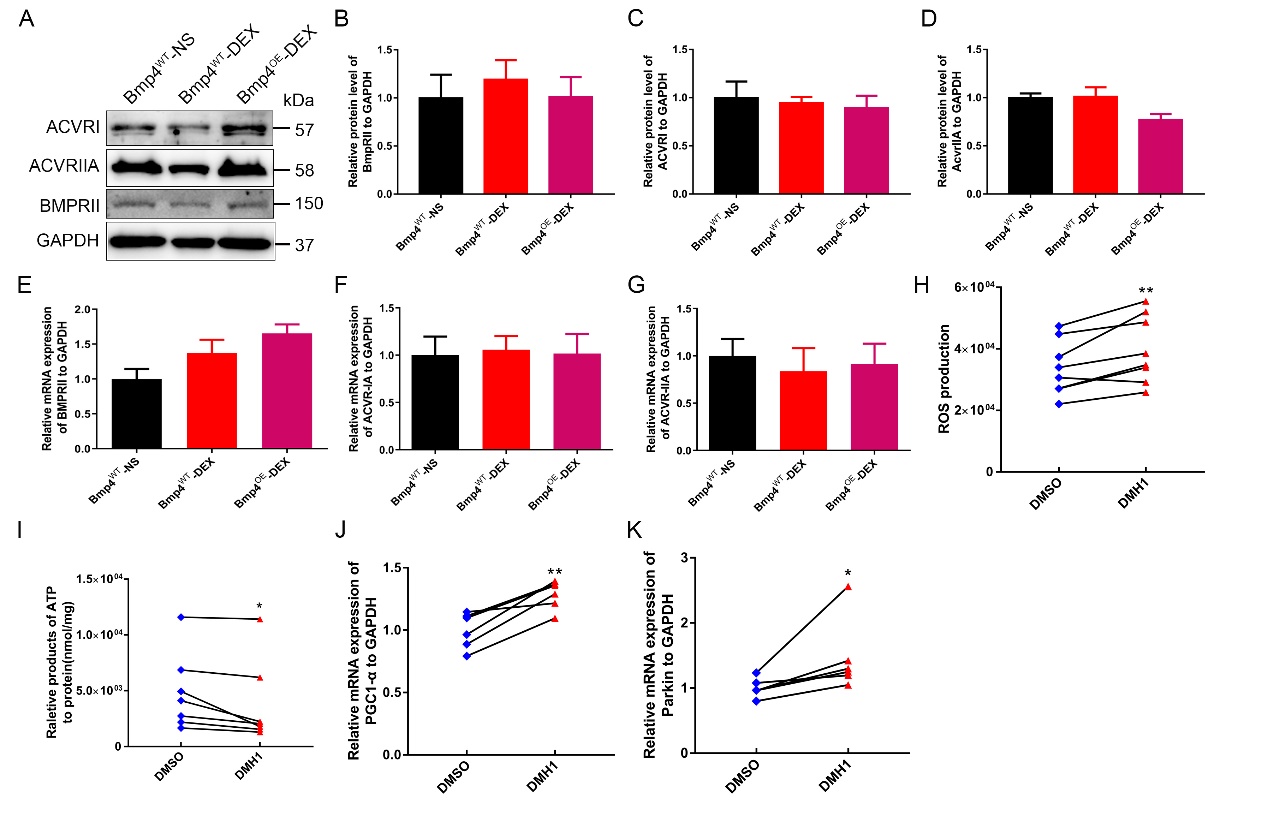


sFig.4: The expression and functions of BMP4 receptors in NMCMs mediating the protective effects of BMP4. The representative immunoblot showing the expression of ACVRI, ACVRIIA and BmpRII in neonatal cardiomyocyte prenatally exposed to NS or DEX (A). Quantitative analysis of ACVRI, ACVRIIA and BmpRII protein expression were shown in B, C and D, respectively (n=6). The mRNA expression levels of ACVRI, ACVRIIA and BmpRII were shown in E, F and G, respectively (n=6). The accumulation of ROS was increased (H), while the ATP level was decreased upon the treatment of specific BmpRII antagonist, DMH1 (I). DMH1 treatment could increase the mRNA expression of PGC-1α and Parkin in NMCMs (J and K). n=6, *p<0.05, **p<0.01, compared with NMCMs treated with vehicle control (DMSO).
